# *IL11^+^* fibroblasts are implicated in nonresponse to anti–TNF-**α** via fibrosis in inflammatory bowel disease

**DOI:** 10.1172/jci.insight.198895

**Published:** 2026-04-23

**Authors:** Wangyue Li, Wei Huang, Jiaxin Wang, Yiwen Tu, Qidi Yang, Yao Zhou, Zile Zhang, Haiming Zhuang, Yubei Gu, Duowu Zou, Yao Zhang

**Affiliations:** Department of Gastroenterology, Ruijin Hospital, Shanghai Jiao Tong University School of Medicine, Shanghai, China.

**Keywords:** Gastroenterology, Inflammation, Fibrosis

## Abstract

Inflammatory bowel disease (IBD) is frequently accompanied by intestinal fibrosis, with nonresponse to long-term anti–TNF-α therapy occurring in approximately 23%–46% of patients. Integrated analysis of single-cell and bulk RNA-seq datasets revealed an expansion of *IL11^+^* fibroblasts in inflamed intestine and their significant enrichment in nonresponders. We further identified *IL11^+^* fibroblasts as a central communication hub that engaged in extensive crosstalk with monocytes and may contribute to inflammatory amplification and fibrotic remodeling. Additionally, we employed machine learning approaches, including least absolute shrinkage and selection operator, support vector machines, and random forest, to derive an *IL11^+^* fibroblast–related gene signature effectively predicting nonresponse to anti–TNF-α in validation and test cohorts. IHC further confirmed the overexpression of IL-11 in nonresponders. The signature genes we found are not only associated with immune and inflammatory responses but also with fibrosis, indicating a robust association between fibrosis and anti–TNF-α treatment failure. In summary, this study highlights the important role of *IL11^+^* fibroblasts in orchestrating both inflammation and fibrosis and provides an applicable model for predicting nonresponse to anti–TNF-α in IBD, thereby laying the foundation for precision medicine and targeted therapeutic strategies.

## Abstract 2

IL11⁺ fibroblasts are associated with fibrosis and anti-TNF non-response in inflammatory bowel disease, identifying IL-11 as a predictive biomarker.

## Introduction

Inflammatory bowel disease (IBD) comprises a group of chronic, idiopathic inflammatory disorders of the gut, primarily classified as Crohn disease (CD) and ulcerative colitis (UC). The global incidence of CD continues to rise, and only 20%–30% of patients experience a nonprogressive or indolent disease course over the long-term (1). UC is associated with high morbidity yet relatively low mortality, whereas patients with longstanding UC carry a substantially increased risk of developing dysplasia and colorectal cancer (2). As a pervasive health challenge, the global age-standardized incidence rate of IBD increased from 4.22 to 4.45 per 100,000 between 1990 and 2021, and its age-standardized mortality rate in the World Bank high-income region also rose by 1.00 during this period (3).

Biologics have been widely used in the management of IBD to induce remission and mitigate complications. Anti–TNF-α agents, such as infliximab (IFX) and adalimumab, are preferred for induction or maintenance therapy in patients refractory to glucocorticoids or azathioprine (1, 4). As a key proinflammatory cytokine, TNF recruits and activates lymphocytes and granulocytes, thereby driving the initiation and progression of intestinal mucosal inflammation in IBD. Anti–TNF-α specifically neutralizes TNF-α, inhibits downstream immune responses, and promotes the apoptosis of inflammatory cells (4, 5). In clinical practice, although anti–TNF-α demonstrates significant efficacy in reducing disease activity, its long-term efficacy is hampered by nonresponse in 23%–46% of patients (6). Nevertheless, the determinants of anti–TNF-α treatment efficacy remain incompletely understood (7).

Intestinal fibrosis, a severe IBD complication driven by inflammation, is characterized by persistent fibroblast activation and pathological extracellular matrix (ECM) deposition (8). Accumulating evidence implicates fibrosis in anti–TNF-α nonresponse. Histological and transcriptomic analyses of intestinal tissues from anti–TNF-α nonresponders reveal elevated fibrosis markers including collagen III and fibronectin, as well as upregulation of related genes like *IL11*, *TNFAIP6*, and *S100A8* (9, 10). Clinical data further indicate that baseline fibrosis severity negatively correlates with anti–TNF-α efficacy, and anti–TNF-α has limited long-term benefits for patients with stricturing CD (11–13). Thus, fibrosis is strongly associated with anti–TNF-α nonresponse, though causality requires clarification. Intestinal fibroblasts are pivotal drivers of fibrosis and key orchestrators of immune microenvironment (8, 14, 15), yet their role in nonresponse to anti–TNF-α remains unclear. Elucidating their function is pivotal, as they may act as a nexus linking fibrosis to anti–TNF-α treatment failure (16).

Meanwhile, several multi-gene models have been proposed to predict anti–TNF-α treatment response (17–19). However, inconsistent definitions of nonresponse, divergent conclusions, and methodological homogeneity limit their generalizability (6, 20–22). Moreover, these models lack biological interpretability, as they do not link predictive gene signatures to specific cellular sources.

By integrating single-cell RNA-seq (scRNA-seq) and bulk RNA-seq, we aim to identify the key fibroblast subcluster exerting profibrotic effects in the inflamed intestine of anti–TNF-α nonresponders and delineate their intercellular interactions. Leveraging machine learning methods including least absolute shrinkage and selection operator (LASSO), support vector machine (SVM), and random forest, we derived gene signatures from *IL11^+^* fibroblasts to construct a predictive model for nonresponse to anti–TNF-α, offering a potential strategy for personalized management in IBD.

## Results

### Single-cell landscape of the inflamed intestine in patients with IBD.

**Figure 1:**
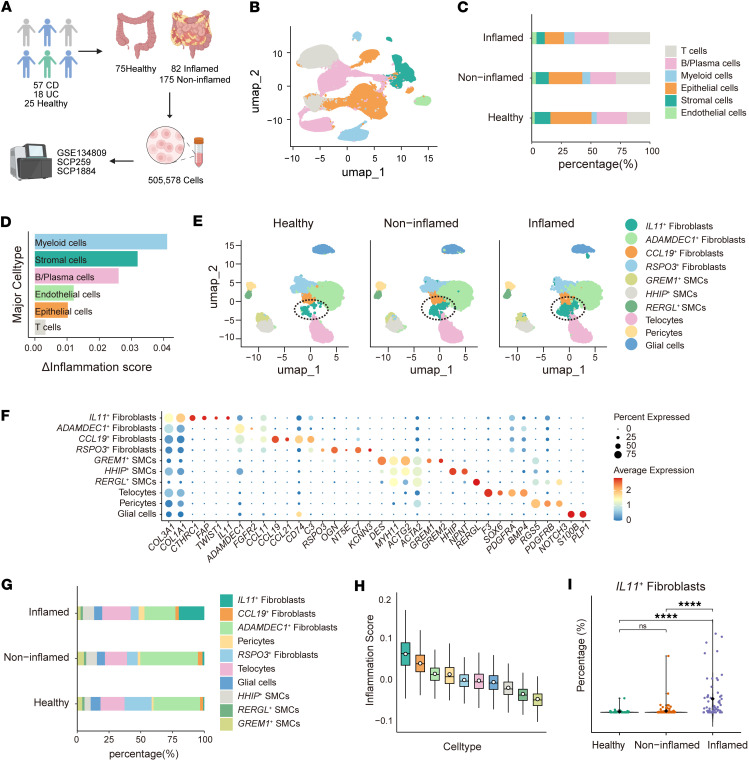
Cellular composition of the inflamed gut in IBD at single-cell resolution. (**A**) Schematic of the integrated scRNA-seq dataset (including GSE134809, SCP259, and SCP1884) comprising 57 patients with CD, 18 with UC, and 25 healthy controls as well as 75 healthy, 175 noninflamed, and 82 inflamed intestinal tissue samples from these patients, where 505,578 cells were identified for analysis. Created with BioRender.com. (**B**) UMAP plot showing 6 intestinal cell subclusters from the integrated scRNA-seq dataset, including 96,139 cells from healthy control group, 286,897 from noninflamed group, and 122,542 from inflamed group, totaling 505,578 cells. (**C**) Bar graph showing the proportions of 6 intestinal cell subclusters across the inflamed, noninflamed, and healthy control groups. (**D**) Changes in the inflammation score (Δ Inflammation score = inflammation score of a subcluster in the inflamed group minus inflammation score of a subcluster in the healthy control group) for each intestinal cell subcluster. (**E**) UMAP plot showing 10 stromal cell subclusters from the integrated scRNA-seq dataset, including 12,555 cells from healthy control group, 30,695 from noninflamed group, and 7,802 from inflamed group, totaling 51,052 cells. (**F**) Dot plot showing the characteristic marker genes for each stromal cell subcluster. Dot color and size represent average expression level and expression percentage, respectively. (**G**) Bar graph showing the proportions of stromal cell subclusters across the inflamed, noninflamed, and healthy control groups. (**H**) Box plots showing the inflammation scores for each stromal cell subcluster. Median, quartiles, and range are shown. (**I**) Proportion of *IL11^+^* fibroblasts in the healthy control, noninflamed, and inflamed groups. Statistical significance was determined by Wilcoxon’s rank-sum test (NS *P* > 0.05; ****P ≤ 0.0001).

To comprehensively investigate the intestinal cellular composition of patients with IBD and its association with inflammation, 3 public scRNA-seq datasets (GSE134809, SCP259, and SCP1884) (16, 23, 24) comprising inflamed, noninflamed, and healthy control intestinal samples were integrated into a new dataset for downstream analysis (Figure 1A). After quality control and initial clustering, a total of 505,578 high-quality cells were retained. Through uniform manifold approximation and projection (UMAP) nonlinear dimensionality reduction, unsupervised clustering, and annotation based on classical marker genes, 6 major intestinal cell types were identified: T cells, B/plasma cells, myeloid cells, epithelial cells, stromal cells, and endothelial cells (Figure 1B). Proportions of T cells, B/plasma cells and myeloid cells were increased in the inflamed group (Figure 1C). To quantify the proinflammatory activity of the 6 identified intestinal cell types, we calculated the change of inflammation score between inflammatory and healthy phenotypes for each subcluster using a published gene set (25). Myeloid cells exhibited the most pronounced score increase under the inflammation condition, followed by stromal cells and B/plasma cells (Figure 1D).

Our previous research (26) revealed that specific mesenchymal stromal cell subclusters, particularly fibroblasts, play important roles in intestinal inflammation and fibrosis in IBD. Therefore, stromal cells were selected for further subclustering and investigation. A total of 51,052 stromal cells were identified, including 12,555 cells from the healthy control group, 7,802 from the inflamed group, and 30,695 from the noninflamed group. These cells were partitioned into 10 subclusters: *IL11^+^* fibroblasts, *ADAMDEC1^+^* fibroblasts, *CCL19^+^* cells, *RSPO3^+^* fibroblasts, *GREM1^+^* smooth muscle cells (SMCs), *HHIP^+^* SMCs, *RERGL^+^* SMCs, telocytes, pericytes, and glial cells (Figure 1E). Their respective marker genes are presented in Figure 1F. Notably, *IL11^+^* fibroblasts were enriched within the inflamed group, whereas they were largely absent in the noninflamed and healthy control groups, suggesting their inflammation-specific expansion (Figure 1G). Given the prominent increase in the inflammation score of myeloid cells (Figure 1D), we likewise subclustered them using an analogous workflow. Supplemental Figure 1, A–C, sequentially displays the UMAP plot of myeloid cells, the proportions of myeloid subclusters across different groups, and their respective marker genes (supplemental material available online with this article; https://doi.org/10.1172/jci.insight.198895DS1).

Subsequent calculation of inflammation scores for each stromal subcluster revealed that *IL11^+^* fibroblasts exhibited the highest proinflammatory activity, followed by *CCL19^+^* fibroblasts and *ADAMDEC1^+^* fibroblasts (Figure 1H). Consistent with this finding, *IL11^+^* fibroblasts were significantly expanded in the inflamed group, whereas their abundance remained unchanged in the noninflamed group compared with the healthy control group (Figure 1I). Among the myeloid subclusters, the previously described inflammation score and an ECM gene signature score based on GO:0030198 from the Gene Ontology (GO) Resource were calculated. *FCGR3A^+^* monocytes and *MMP9^+^* macrophages exhibited the highest inflammation and ECM scores, respectively, and *S100A8/9^+^* monocytes ranked second in both scores (Supplemental Figure 1, D and E). These 3 subclusters were also significantly enriched in the inflamed group (Supplemental Figure 1F). Together, these findings indicate that *IL11^+^* fibroblasts as well as specific myeloid subclusters such as *S100A8/9^+^* monocytes and *FCGR3A^+^* monocytes expand under the inflammation condition and possess high proinflammatory activity, indicating their potentially important roles in the pathogenesis of IBD, especially CD.

### An IL11^+^ fibroblast–centric cell module plays a significant role in inflammation.

**Figure 2:**
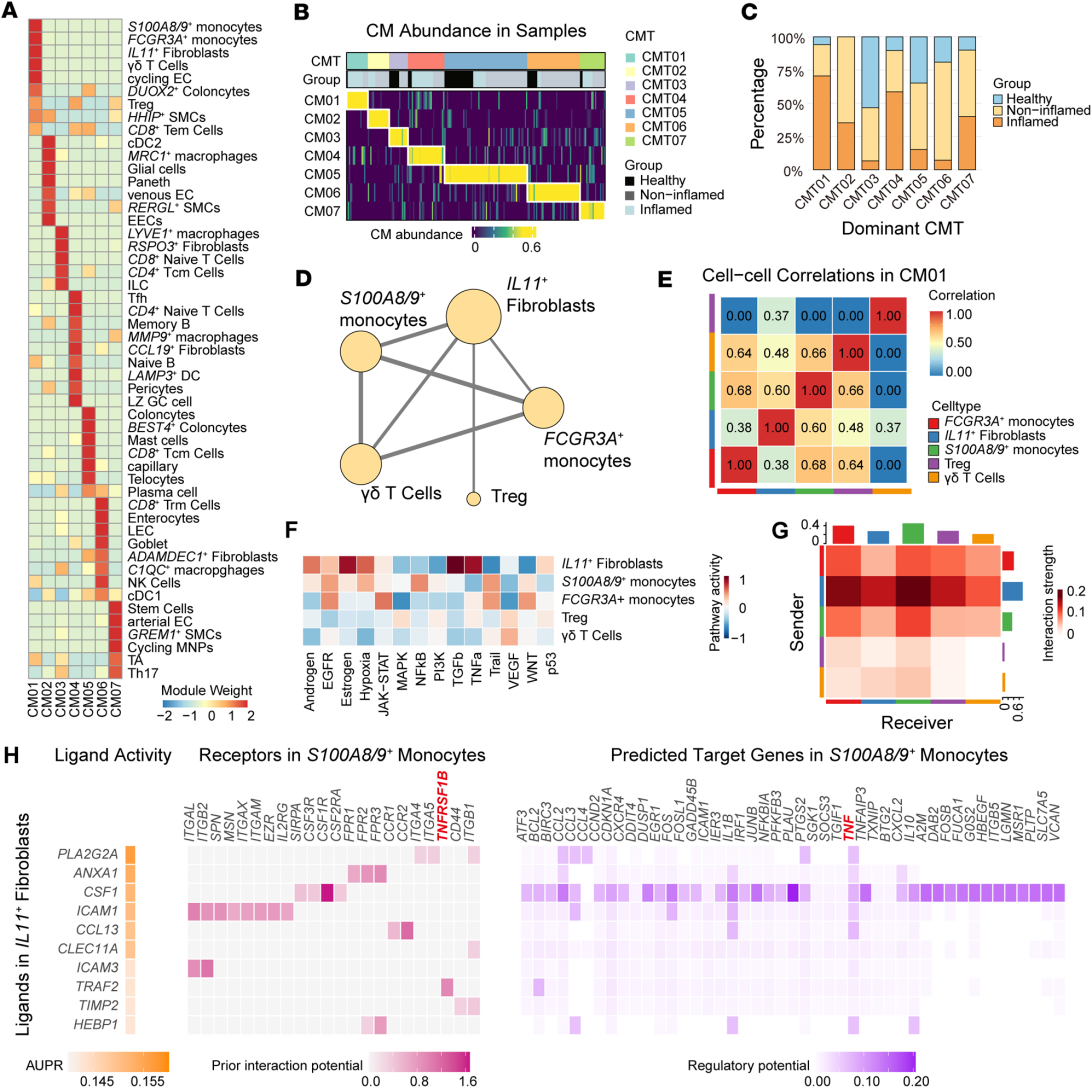
An *IL11^+^* fibroblast–centric CM and its intercellular interaction network. (**A**) Heatmap showing the cellular composition of CM01-07. Grid color represents the module weight of each subcluster across different CMs. (**B**) Abundance of each CM across CMTs and groups. The color codes for each CMT and group are listed on the right. Grid color represents the abundance of CMs in each sample. (**C**) Bar graph showing the proportions of the healthy control, noninflamed, and inflamed samples within each CMT. (**D**) Network graph showing associations among the core subclusters of CM01 (*IL11^+^* fibroblasts, *S100A8/9^+^* monocytes, *FCGR3A^+^* monocytes, Tregs, and γδ T cells). Node size represents the number of associations, and line thickness represents the association strength. (**E**) Heatmap showing Spearman’s correlations among the core subclusters of CM01. The color scale for correlation coefficients and subcluster annotations are shown on the right. (**F**) Heatmap showing the activity of PROGENy-annotated inflammation-associated pathways in the 5 core subclusters of CM01. (**G**) Heatmap showing the interaction strength among the 5 core subclusters of CM01. (**H**) Heatmap displaying the top-ranked ligand-receptor pairs inferred by NicheNet for *IL11^+^* fibroblasts regulating *S100A8/9^+^* monocytes. The left panel shows the activity of top-ranked ligands from *IL11^+^* fibroblasts and their interaction strength with potential receptors on *S100A8/9^+^* monocytes; the right panel shows the downstream target genes in *S100A8/9^+^* monocytes. The meaning represented by different grid colors is shown at the bottom of the figure.

A cell module (CM) is a coordinated multicellular ecosystem wherein diverse types of cells collectively interact and function in a cohesive manner (27). To identify CMs closely associated with inflammation and internal interaction patterns within a single module, CoVarNet, an algorithm that infers multi-cell co-occurrence and synergistic modules (27), was applied for further analysis of the scRNA-seq dataset. By executing a standard computational workflow with precise parameter selection (Supplemental Figure 2A), 7 CMs stably present in intestine were identified, with *IL11^+^* fibroblasts assigned to CM01 (Figure 2A). Subsequently, analysis of CM types (CMTs) — sample sets enriching in corresponding CMs — revealed that CMT01 showed the strongest association with inflammation and contained the highest proportion of inflamed samples among all CMTs (Figure 2, B and C). Therefore, the cellular interaction network within CM01 was selected as the focus for further investigation. Pearson’s correlation–based edge analysis identified *IL11^+^* fibroblasts, *S100A8/9^+^* monocytes, *FCGR3A^+^* monocytes, γδ T cells, and Tregs as central hubs within CM01 (Figure 2D). Among these, *S100A8/9^+^* monocytes possessed the highest weight, and *IL11^+^* fibroblasts exhibited the highest degree (number of associations with other subclusters in the module) (Supplemental Figure 2, B and C). Notably, *IL11^+^* fibroblasts were also among the highest-degree subclusters in the global interaction network (Supplemental Figure 2D). Furthermore, cell proportion correlation analysis showed strong covariation among *IL11^+^* fibroblasts, *S100A8/9^+^* monocytes, and *FCGR3A^+^* monocytes (Figure 2E).

To further elucidate the signaling pathways and molecular basis underlying these cellular interactions, downstream analyses based on PROGENy (28), CellChat (29), and NicheNet (30) were performed. Among central subclusters in CM01, *IL11^+^* fibroblasts exhibited markedly high activity in TNF-α and TGF-β signaling pathways (Figure 2F). Within the cellular communication network of CM01, *IL11^+^* fibroblasts predominantly acted as signal senders, interacting extensively with *S100A8/9^+^* monocytes and *FCGR3A^+^* monocytes, particularly with the former (Figure 2G and Supplemental Figure 2E). Ligand-receptor-target inference predicted that *IL11^+^* fibroblasts exhibited high activity of ligands including *PLA2G2A*, *ANXA1*, *CSF1*, and *TRAF2*, potentially interacting with receptors such as *ITGA*, *FPR*, *CSF1R*, and especially *TNFRSF1B* — a gene previously associated with anti–TNF-α nonresponse (24) — thereby promoting the expression of downstream proinflammatory and profibrotic genes including *TNF* (Figure 2H). Supplemental Figure 2F further indicates that *S100A8/9^+^* monocytes serve as the major cellular source of TNF pathway signaling within CM01. Additionally, *IL11^+^* fibroblasts exert a potential regulatory effect on *IL1B* expression in *FCGR3A^+^* monocytes (Supplemental Figure 2G). Collectively, the *IL11^+^* fibroblast–centered CM01 showed a strong correlation with inflammatory phenotype, wherein *IL11^+^* fibroblasts closely interact with *S100A8/9^+^* monocytes and *FCGR3A^+^* monocytes to participate in inflammation.

### IL11^+^ fibroblasts are closely linked to a profibrotic role.

**Figure 3:**
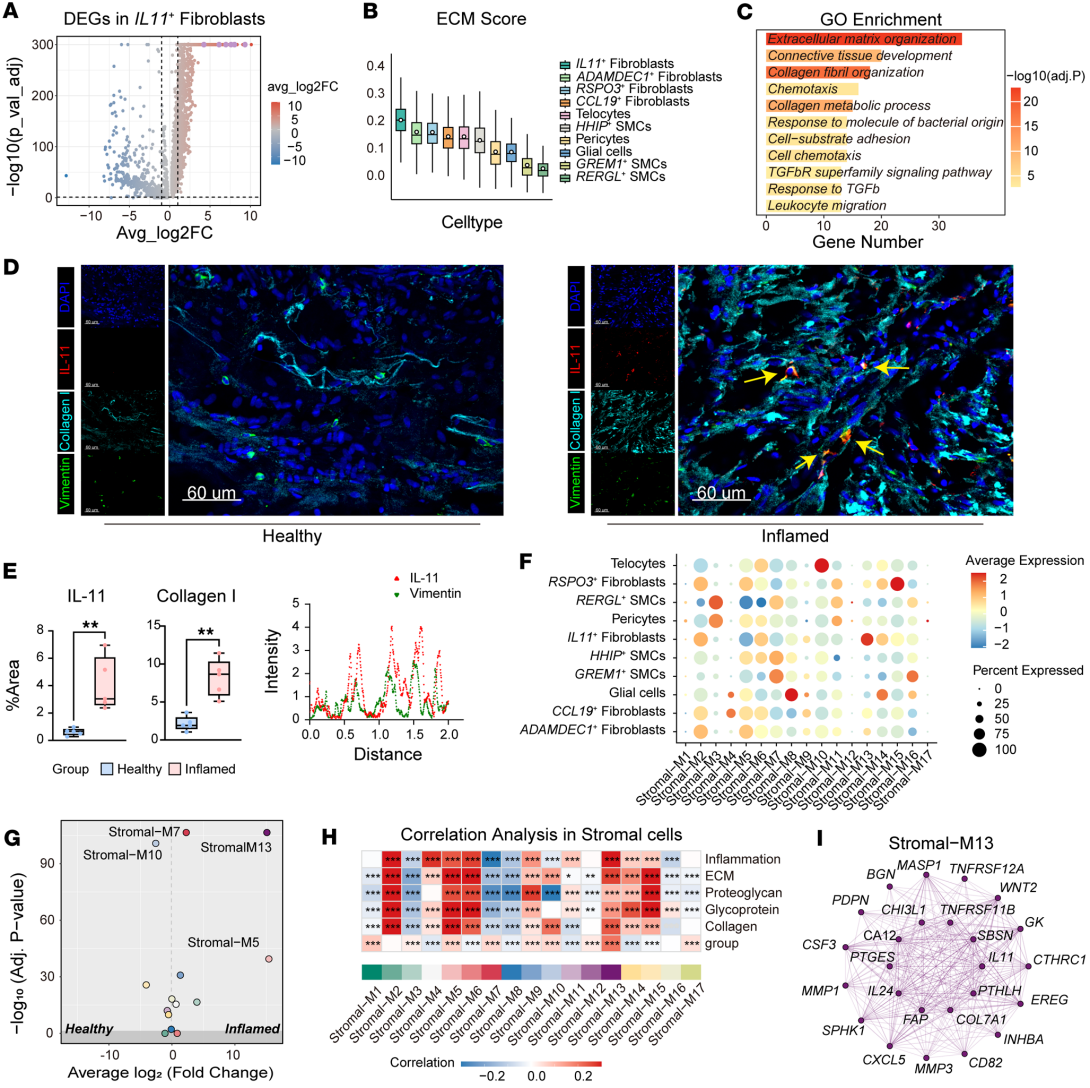
*IL11^+^* fibroblasts exhibit a profibrotic transcriptional program. (**A**) Volcano plot of DEGs in *IL11^+^* fibroblasts versus other stromal cells in the inflamed group. Red and blue dots represent significantly upregulated and downregulated genes, respectively (adjusted *P* < 0.05; average log_2_ fold change > 4). (**B**) Box plots showing the ECM scores for each stromal cell subcluster. Median, quartiles, and range are shown. (**C**) Representative GO enrichment analysis for the DEGs of *IL11^+^* fibroblasts. A hypergeometric test was conducted using FDR-adjusted *P* values. (**D**) Representative immunofluorescence images of surgically resected human intestinal segments including inflamed and healthy area. Merged channels showing DAPI (blue), IL-11 (red), collagen I (cyan), and vimentin (green) are displayed. Yellow arrows indicate *IL11^+^* fibroblasts. Original magnification: ×20. Scale bar: 60 μm. The experiment was conducted in 5 inflamed and 5 healthy tissues. (**E**) Left: box plots showing the expression of IL-11 and collagen I in inflamed and healthy intestinal tissues. Median, quartiles, and range are shown. Statistical significance was determined by Wilcoxon’s rank-sum test (**P ≤ 0.01); right: distance-intensity colocalization profile of IL-11 and vimentin in the same tissue samples. (**F**) Dot plot showing the expression of stromal-M1 to -M13 across stromal cell subclusters. Dot color and size represent average expression level and expression percentage, respectively. (**G**) Differential expression of gene modules between the inflamed and healthy control groups. Stromal-M1, -M12, and -M17 were excluded due to low expression levels. (**H**) Heatmap showing Pearson’s correlations and its significance between stromal-M1 to M17 and inflammation/ECM/proteoglycan/glycoprotein/collagen scores, as well as group phenotypes (inflamed/noninflamed/healthy control). Statistical significance was determined by Wilcoxon’s rank-sum test with Benjamini-Hochberg procedure (*P ≤ 0.05; **P ≤ 0.01; ***P ≤ 0.001). (**I**) Association network of the top 25 genes ranked by kME in descending order within stromal-M13. The inner circle highlights the top 10 genes.

Given the observed close association of *IL11^+^* fibroblasts with inflammation, the functional characteristics of their differentially expressed genes (DEGs) were investigated. First, the DEGs of *IL11^+^* fibroblasts within the inflamed group were extracted, among which 180 genes were significantly upregulated (Figure 3A). The ECM score was calculated across stromal cell subclusters, with *IL11^+^* fibroblasts attaining the highest score (Figure 3B). GO enrichment analysis showed that these genes were enriched in fibrosis-related pathways such as ECM organization, connective tissue development, and collagen fibril organization (Figure 3C). Immunofluorescence confirmed that *IL11^+^* fibroblasts were localized to regions of collagen I deposition (Figure 3D). Corresponding quantitative and colocalization analyses further confirmed the coordinate upregulation of IL-11 and collagen I in inflamed intestine, together with the widespread presence of *IL11^+^* fibroblasts (Figure 3E).

Subsequently, high-dimensional weighted gene coexpression network analysis, a method for constructing weighted gene coexpression networks from high-dimensional gene expression data (31), was employed to identify gene modules coexpressed with *IL11^+^* fibroblasts and their functional characteristics. Within the stromal cells in the integrated scRNA-seq dataset, 17 gene modules were constructed. Figure 3F and Supplemental Figure 3A display the module eigenvalue expression levels for each stromal cell subcluster and the top 5 genes of each gene module, respectively, sorted by eigengene-based connectivity (kME), a parameter assessing gene-module correlation. Notably, *IL11^+^* fibroblasts showed high abundance and specificity within stromal-M13, where *IL11* was the top-ranked gene. We next assessed the module differential expression between the inflamed and healthy control groups, excluding modules with low overall expression (stromal-M1, -M12, and -M17) (Figure 3F). The results showed that stromal-M5, -M7, and -M13 were significantly upregulated in the inflamed group, with stromal-M13 showing the most pronounced upregulation, whereas stromal-M10 was significantly downregulated (Figure 3G). Further analysis revealed that stromal-M13 was highly correlated with group phenotypes and showed significantly positive correlations with both inflammation and ECM scores (Figure 3H). Consistently, stromal-M13 was also functionally enriched in ECM-related pathways (Supplemental Figure 3B).

Finally, the gene composition of stromal-M13 was parsed to identify hub genes playing critical roles. Figure 3I shows the association network of the top 25 genes in stromal-M13 ranked by kME, with the inner circle representing the top 10 genes. Notably, *IL11* occupied a central position, with extensive connections to other genes in the network. Moreover, genes implicated in fibrosis-related pathways, including *CHI3L1* and *FAP* (26, 32), also ranked among the top 10 genes. In summary, on the basis of stromal-M13, *IL11^+^* fibroblasts may contribute to the fibrotic process in IBD, especially CD.

### A close association between IL11^+^ fibroblasts and nonresponse to anti–TNF-α.

**Figure 4:**
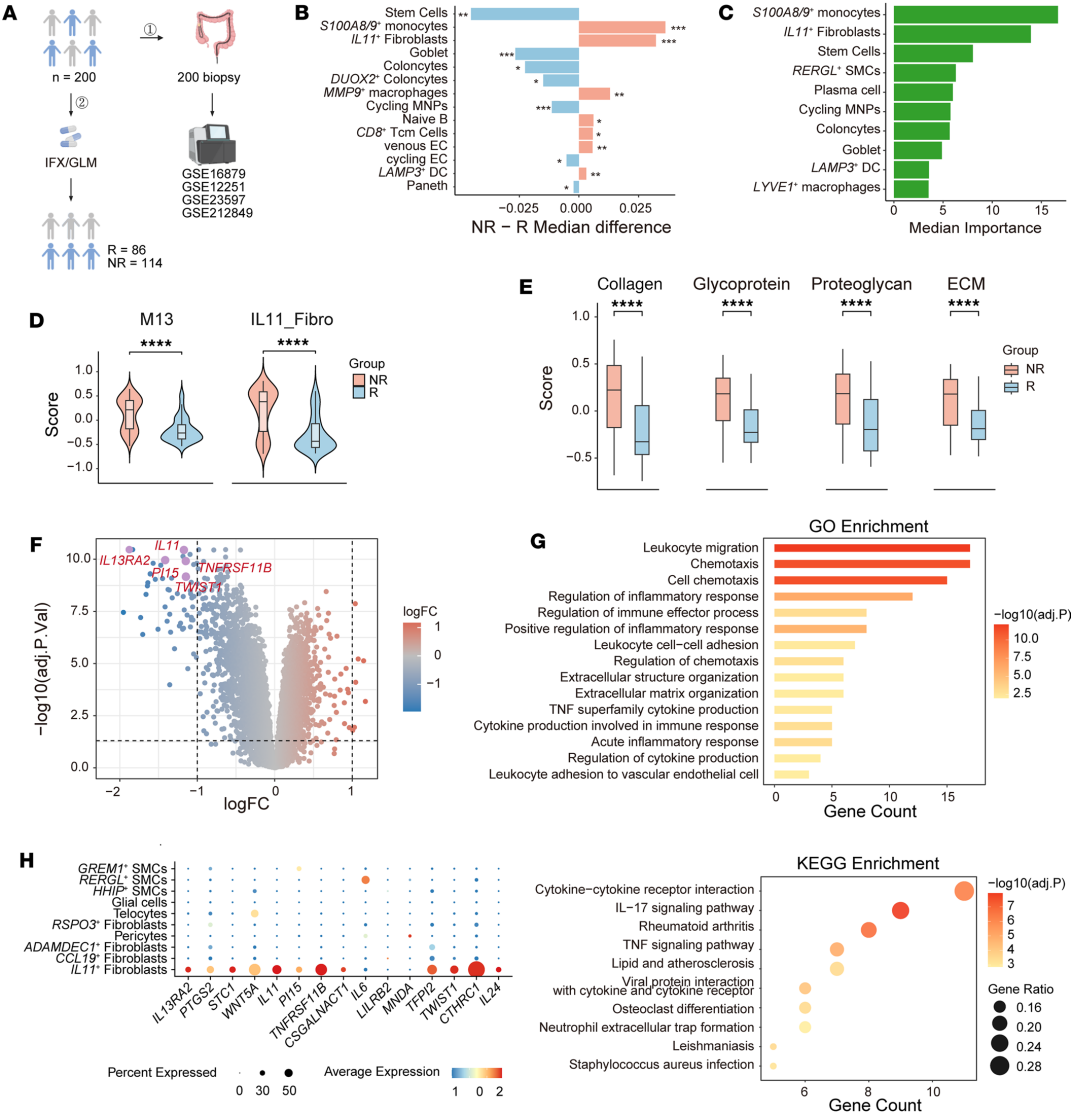
*IL11^+^* fibroblasts are associated with nonresponse to anti–TNF-α. (**A**) Schematic of the integrated bulk RNA-seq dataset (including GSE16879, GSE12251, GSE23597, and GSE212849), comprising intestinal samples from 200 patients with IBD treated with IFX or GLM (86 responders, 114 nonresponders). All samples were obtained prior to IFX or GLM treatment. Created with BioRender.com. (**B**) Median differences in the inferred cell proportions estimated by CIBERSORTx between nonresponders and responders. Statistical significance was determined by Wilcoxon’s rank-sum test with Benjamini-Hochberg procedure (*P ≤ 0.05; **P ≤ 0.01; ***P ≤ 0.001). (**C**) Ranking of nonresponse-associated cell importance based on the Boruta algorithm. (**D** and **E**) ssGSEA scores for nonresponders and responders on the stromal-M13 gene set and the *IL11^+^* fibroblast DEGs (**D**), and on the collagen, glycoprotein, proteoglycan, and ECM gene sets (**E**). Statistical significance was determined by Wilcoxon’s rank-sum test (****P ≤ 0.0001). (**F**) Volcano plot showing differential gene expression between responders and nonresponders. Blue and red dots represent significantly downregulated and upregulated genes, respectively (FDR < 0.05; |log fold change| > 1). (**G**) GO biological process enrichment analysis and KEGG enrichment analysis of nonresponse-associated DEGs. (**H**) Dot plot showing the expression of the top 15 nonresponse-associated DEGs in stromal cell subclusters identified in the integrated scRNA-seq dataset. Dot color and size represent average expression level and expression percentage, respectively.

Since the high *TNF* regulatory activity of *IL11^+^* fibroblasts has been revealed (Figure 2, F and H, and Supplemental Figure 2F), the relationship between these cells and the efficacy of anti–TNF-α therapy deserves further in-depth investigation. We integrated 4 public bulk RNA-seq datasets, GSE16879, GSE12251, GSE23597, and GSE212849 (17, 33–35), comprising pretreatment intestinal tissue samples from 200 patients with IBD treated with IFX or golimumab (GLM), including 86 responders and 114 nonresponders (Figure 4A). Cell type identification by estimating relative subsets of RNA transcripts (CIBERSORTx) (36) was used to infer the cellular composition of the integrated bulk RNA-seq dataset. Results showed that *IL11^+^* fibroblasts and *S100A8/9^+^* monocytes were both significantly enriched in nonresponders (Figure 4B and Supplemental Figure 4A). However, in an independent cohort treated with vedolizumab (VDZ) from another bulk RNA-seq dataset E-MTAB-7914 (including 35 nonresponders, 27 responders) (37), we did not observe significant enrichment of these 2 cell populations in nonresponders (Supplemental Figure 4B). Based on the inferred cell proportions, the R package Boruta (38) was used to rank the importance of various cell subclusters in nonresponse. *S100A8/9^+^* monocytes ranked first, followed by *IL11^+^* fibroblasts (Figure 4C and Supplemental Figure 4B). Notably, the enrichment and synergy of *IL11^+^* fibroblasts and *S100A8/9^+^* monocytes in inflamed intestine had been previously identified in the analysis on the scRNA-seq dataset (Figure 1I, Supplemental Figure 1F, and Figure 2D), suggesting their potential broad interaction in both inflammation and nonresponse.

Given the confirmed association between nonresponse and *IL11^+^* fibroblasts — a subcluster with profibrotic features (Figure 3) — attention was turned to whether nonresponse might also be associated with fibrosis. Therefore, single sample gene set enrichment analysis (ssGSEA) was used to calculate scores in gene sets derived from *IL11^+^* fibroblasts and stromal-M13, as well as published collagen/glycoprotein/proteoglycan-related gene sets (39) and the ECM gene set used in previous analysis. Nonresponders showed elevated scores in both the stromal-M13 and *IL11^+^* fibroblast–derived gene sets (Figure 4D), consistent with previous results of cell type identification (Figure 4, B and C). Moreover, nonresponders showed significantly higher enrichment of collagen-, glycoprotein-, proteoglycan-, and ECM-related gene sets compared with responders (Figure 4E).

To further identify genes closely associated with nonresponse, limma (40) was used to compare the gene expression between nonresponders and responders, screening out 60 DEGs. Figure 4F shows that *IL13RA2*, *PI15*, *IL11*, *TNFRSF11B*, and *TWIST1* were significantly upregulated in nonresponders. GO and Kyoto Encyclopedia of Genes and Genomes (KEGG) enrichment analyses performed on all 60 DEGs showed enrichment in inflammation- and fibrosis-related pathways (Figure 4G), and the top 15 DEGs were also enriched in *IL11^+^* fibroblasts (Figure 4H). Consistent with the above evidence, previous studies have reported an association between intestinal fibrosis and nonresponse (9, 10, 24, 34, 41). Thus, fibrosis may constitute a mechanistic link between *IL11^+^* fibroblasts and nonresponse to anti–TNF-α.

### An IL11^+^ fibroblast–associated multi-gene model predicting nonresponse to anti–TNF-α.

**Figure 5:**
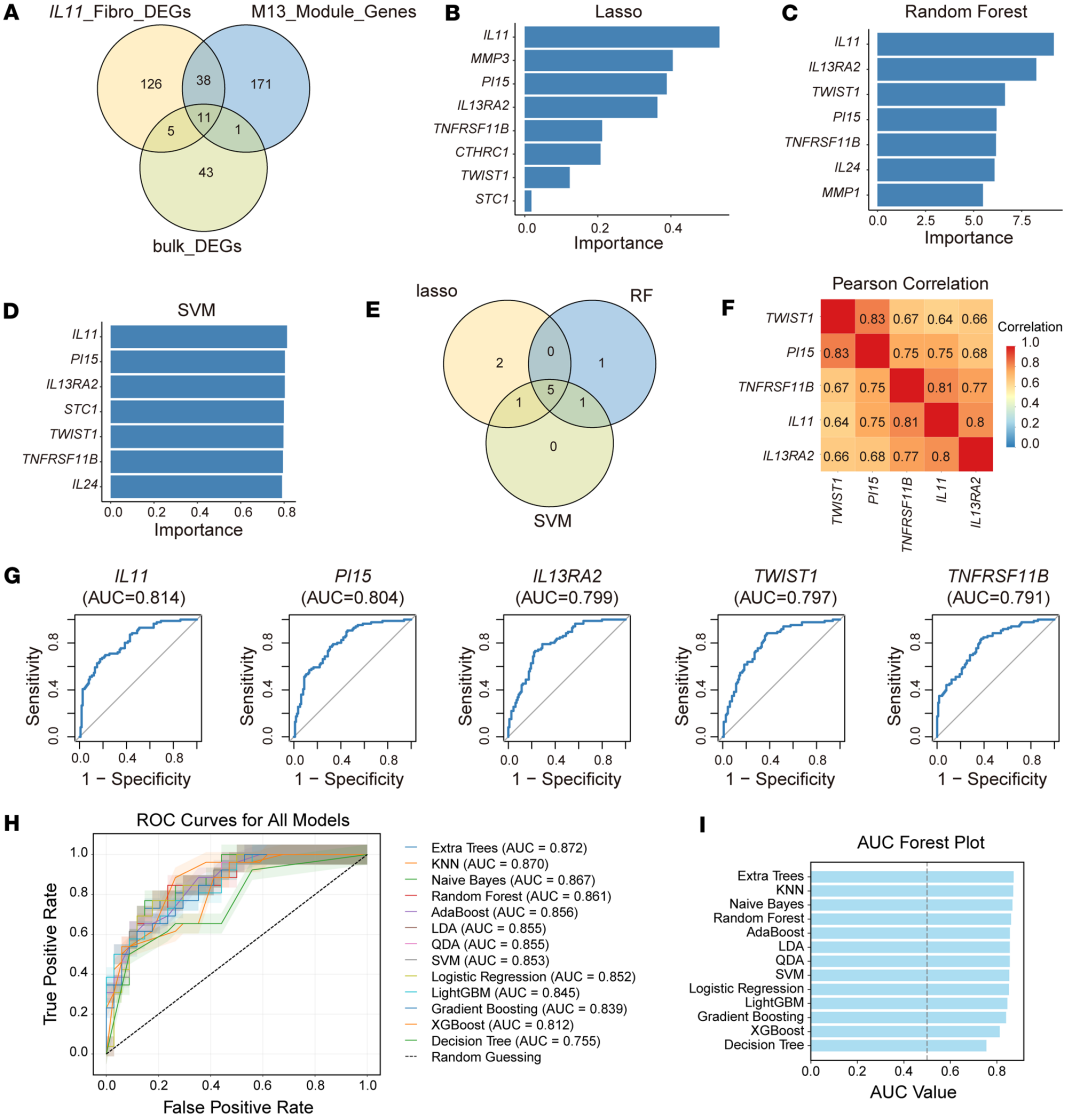
Development of a predictive model for nonresponse to anti–TNF-α based on *IL11^+^* fibroblasts. (**A**) Venn diagram depicting the overlap among *IL11^+^* fibroblast DEGs, genes in stromal-M13, and nonresponse-associated genes. (**B**–**D**) Feature selection by LASSO (**B**), random forest (**C**), and SVM (**D**). The optimal factor sets selected by each algorithm are shown in vertical axes. (**E**) Venn diagram showing the intersection of factor sets identified by LASSO, random forest, and SVM, finally yielding 5 key genes. (**F**) Heatmap showing Pearson’s correlation coefficients among the key genes (*TWIST1*, *PI15*, *TNFRSF11B*, *IL11*, *IL13RA2*). Statistical significance was determined by Wilcoxon rank-sum test with Benjamini-Hochberg procedure. (**G**) ROC curves of *TWIST1*, *PI15*, *TNFRSF11B*, *IL11*, and *IL13RA2* in predicting nonresponse to anti–TNF-α. (**H**) ROC curves of the nonresponse prediction models constructed by applying 13 machine learning algorithms to the key genes. Used algorithm and corresponding model AUC values are listed on the right. (**I**) Forest plot showing AUC values of all 13 prediction models.

Given the association between *IL11^+^* fibroblasts and nonresponse to anti–TNF-α, whether gene characteristics of these cells could predict anti–TNF-α treatment outcomes warranted further exploration. First, 11 candidate genes overlapping between DEGs of *IL11^+^* fibroblasts (Figure 3A), genes in stromal-M13, and nonresponse-associated genes (Figure 4F) were identified (Figure 5A). LASSO, random forest, and SVM were employed to screen gene signatures for constructing the predictive model. Figure 5, B–D, and Supplemental Figure 5, A–C, illustrate the optimal composition of gene signatures identified by the 3 machine learning algorithms.

Subsequently, 5 key genes including *IL11*, *PI15*, *IL13RA2*, *TWIST1*, and *TNFRSF11B* were pinpointed from the intersection of LASSO, random forest, and SVM analyses (Figure 5E). Interestingly, all these key genes were significantly upregulated in nonresponders (Figure 4F) and have been previously implicated in both inflammation and fibrosis (24, 26, 42, 43). In addition, we performed qPCR on paired fibrotic and nonfibrotic intestinal samples from 8 patients with CD and found that these key genes were significantly upregulated in fibrotic segments (Supplemental Figure 5D), further supporting their association with fibrosis. Pearson’s correlation analysis showed that these genes were highly correlated, with correlation coefficients ranging from 0.66 to 0.83 (Figure 5F). Moreover, receiver operating characteristic (ROC) analysis for each key gene yielded AUCs ranging from 0.791 to 0.814, indicating their potential as predictors for nonresponse to anti–TNF-α (Figure 5G).

Finally, 13 commonly used machine learning modeling methods including k-nearest neighbor (KNN), random forest, AdaBoost, Extra Trees, SVM, logistic regression, linear discriminant analysis (LDA), quadratic discriminant analysis (QDA), naive Bayes, LightGBM, Gradient Boosting, XGBoost, and Decision Tree were applied to construct nonresponse prediction models based on the aforementioned 5 key genes, with the integrated bulk RNA-seq dataset being randomly split into a training set (*n* = 140) and a validation set (*n* = 60). ROC curves demonstrated that the AUCs for all models in the validation set were no less than 0.755, with the Extra Trees model showing the best performance (AUC = 0.872) (Figure 5, H and I). The Extra Trees model also exhibited balanced sensitivity, specificity, and accuracy compared with other models (Supplemental Figure 5E). Collectively, these data indicate that the *IL11^+^* fibroblast–derived gene signatures can effectively predict response to anti–TNF-α in IBD.

### IL11 serves as a key factor in the nonresponse prediction model.

**Figure 6:**
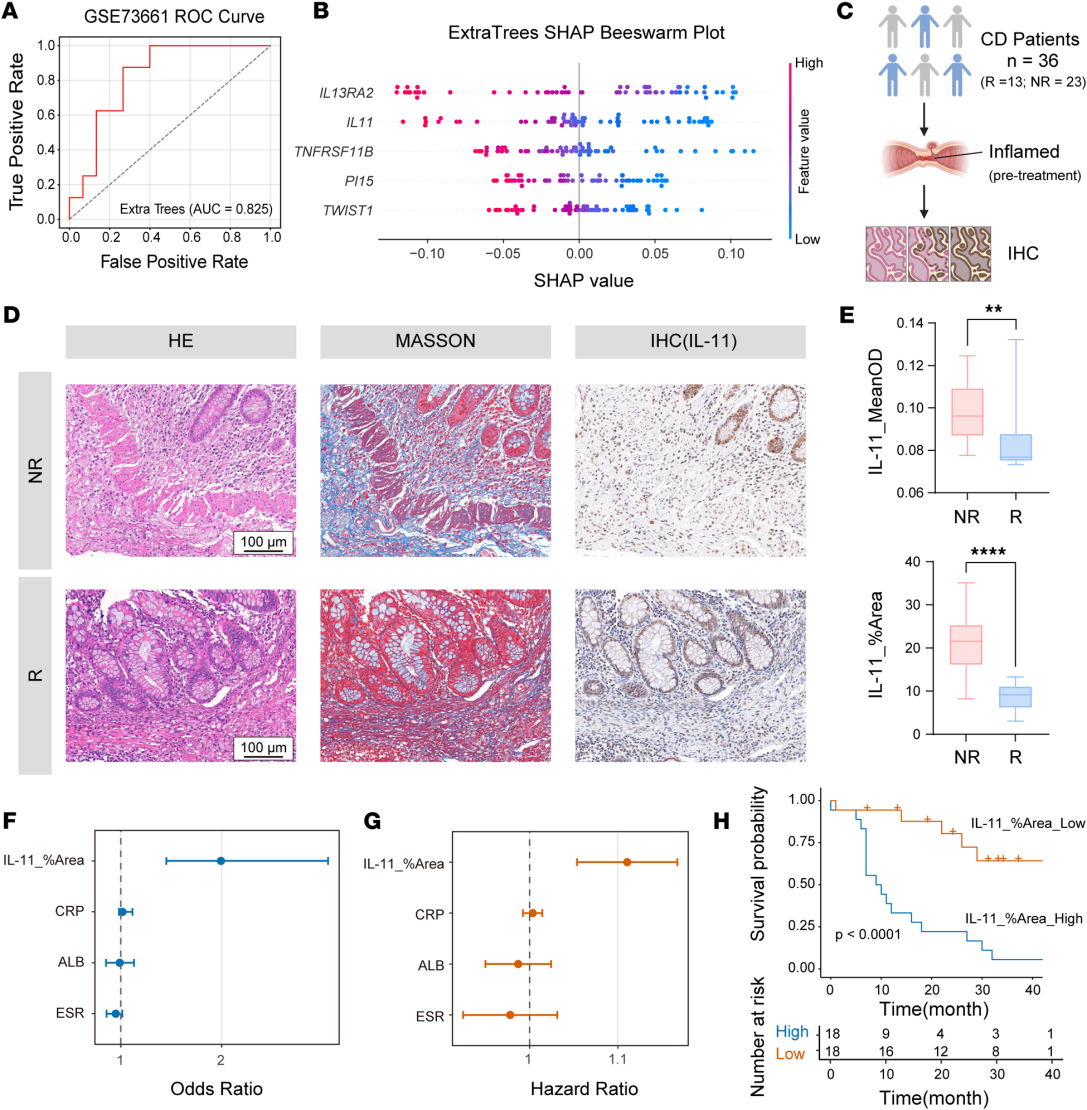
The central role of *IL11* in the nonresponse prediction model. (**A**) ROC curve showing the predictive performance of the nonresponse prediction model in the independent test set GSE73661 (AUC = 0.825). (**B**) Bee-swarm plot showing the contribution strength and direction of each gene in the model to the prediction value. Dot color reflects feature values (red: high; blue: low). (**C**) Graphic overview illustrating the source and composition of samples for staining experiments including IHC. Pretreatment inflamed bowel samples were obtained from 36 patients with CD, comprising 13 responders and 23 nonresponders. Created with BioRender.com. (**D**) Representative images of H&E, Masson’s trichrome, and IL-11 IHC staining of inflamed intestine sections from a nonresponder and a responder. Original magnification: ×10. Scale bar: 100 μm. These experiments were conducted in 23 nonresponders and 13 responders. (**E**) Box plots showing the mean OD of IL-11–stained area and IL-11_%Area (the percentage of IL-11–positive area) of nonresponders (*n* = 23) and responders (*n* = 13). Median, quartiles, and range are shown. Statistical significance was determined by Wilcoxon’s rank-sum test (**P ≤ 0.01; ****P ≤ 0.0001). (**F** and **G**) Forest plots showing the ORs (**F**) and HRs (**G**) of IL-11_%Area, CRP, ALB, and ESR for the occurrence of nonresponse in the cohort. (**H**) Probability change of biologic treatment response over time in patients stratified by high/low IL-11_%Area (based on the median value of IL-11_%Area). Top: Kaplan-Meier survival curves for the 2 groups; bottom: number of patients at risk within each group. Statistical significance was determined by the log-rank test.

To assess the robustness of our model, we applied it to an independent test set (GSE73661) comprising 15 nonresponders and 8 responders with UC (44). Figure 6A shows that the model yielded an AUC of 0.825 in this dataset, comparable to its performance in the validation set (Figure 5I). In addition, we calibrated 3 previously published predictive models for anti–TNF-α response in IBD, designated as models 1 (18), 2 (17), and 3 (34), in the training set and compared their performance with that of our model in the validation set. Models 1, 2, and 3 achieved AUC values of 0.824, 0.606, and 0.856, respectively (Supplemental Figure 6A), whereas our Extra Trees model showed superior performance (AUC = 0.872) (Figure 5H). Subsequently, Shapley additive explanations (SHAP) analysis was applied to assess the magnitude and direction of each gene’s contribution to the model’s predictions. All 5 genes showed negative SHAP values, indicating that higher expression of them was associated with an increased probability of nonresponse (Figure 6B).

Given the high contribution of *IL11* to nonresponse prediction and its previously elucidated profibrotic role, its expression level and location in intestine, along with its potential clinical implications, attracted our focus. Pretreatment inflamed bowel samples were obtained from 36 patients with CD, including 13 responders and 23 nonresponders, for IHC staining and other staining experiments (Figure 6C). Compared with responders, nonresponders showed increased collagen deposition and IL-11 expression in lesional tissues (Figure 6, D and E). We then quantified IL-11 staining as the percentage of positive area (IL-11_%Area) and compared it with classic CD inflammatory markers (1) including C-reactive protein (CRP), albumin (ALB), and erythrocyte sedimentation rate (ESR) regarding the OR and HR for the occurrence of nonresponse. Figure 6, F and G, demonstrate that IL-11_%Area showed an OR of 1.99 (95% CI: 1.37–4.16) and an HR of 1.11 (95% CI: 1.05–1.17), outperforming CRP, ALB, and ESR (detailed information is in the Supporting Data Values file). Next, we performed ROC analysis on IL-11_%Area, CRP, ALB, and ESR, as well as lifestyle factors including BMI and smoking, and found that IL-11_%Area emerged as the strongest predictor of nonresponse (AUC = 0.977) (Supplemental Figure 6B). Kaplan-Meier analysis of the same cohort further showed that patients with high IL-11_%Area had a significantly higher incidence of nonresponse than those with low IL-11_%Area (Figure 6H). Together, the *IL11^+^* fibroblast–associated predictive model for nonresponse to anti–TNF-α is generalizable and interpretable, with its core component *IL11* being highly expressed in fibrotic lesions of nonresponders to anti–TNF-α and correlating with response to anti–TNF-α in patients with CD.

## Discussion

Herein, we identified *IL11^+^* fibroblasts as a key cellular component within the inflamed intestine of patients with IBD, where they exert distinct profibrotic roles. Concurrently, we observed significantly enhanced expression of fibrosis-related genes and elevated ECM deposition in the intestinal tissues of anti–TNF-α nonresponders, as well as a fibrosis-related gene module (stromal-M13) demonstrating a valid association with *IL11^+^* fibroblasts. Based on these findings, we developed a multi-gene model for predicting nonresponse to anti–TNF-α, providing a reference for exploring the mechanisms and advancing the clinical practice of precision medicine in IBD.

We first conducted a comprehensive analysis of an integrated scRNA-seq dataset utilizing methods including CoVarNet (27), CellChat (29), and decoupleR (45), revealing an enrichment of *IL11^+^* fibroblasts in the inflamed intestine. These fibroblasts closely interact with inflammatory cells, such as *S100A8/9^+^* monocytes, forming a CM that mediates inflammation via cytokines including TNF, within which *IL11^+^* fibroblasts act as a central hub. IL-11 is consistently upregulated across fibroinflammatory diseases such as IBD, systemic sclerosis, and rheumatoid arthritis, where it drives myofibroblast activation and inflammation through a noncanonical signaling pathway (42, 46, 47). In a recent study on intestinal fibrosis, Pokatayev et al. (47), utilizing scRNA-seq combined with spatial transcriptomics, revealed that inflammation-associated fibroblasts (IAFs) can be induced by *FCN1^+^IL1B^+^* proinflammatory macrophages to secrete IL-11, thereby exerting profibrotic effects. Although isolated studies have suggested that IL-11 exhibits a cytoprotective effect on mucosal epithelium in dextran sodium sulfate–induced murine colitis, this may be attributed to differences in disease course between acute inflammatory models and chronic inflammation in IBD, as well as cross-species heterogeneity (48). Our findings align with a growing body of evidence highlighting *IL11^+^* fibroblasts in inflammation and cancer. Smillie et al. (24) identified a marked expansion of IAFs exemplified by *IL13RA2^+^IL11^+^* inflammatory fibroblasts within inflamed areas via constructing a cellular atlas of the UC intestine. A recent study employing homologous methodologies also found that *IL11^+^MMP1^+^CXCL8^+^IL7R^+^* inflammatory myofibroblasts, which may recruit neutrophils, monocytes, and B cells, are enriched in inflamed tissues from various diseases including IBD (49). Additionally, Nishina et al. (50) demonstrated that *IL11^+^* fibroblasts activate tumor cells through IL-11 secretion in colorectal cancer and are associated with reduced recurrence-free survival. Notably, Arijs et al. (9) observed upregulation of *IL11* and *S100A8/9* in CD, but the underlying connection between them was not explored, and our study provides a cellular framework for their coexpression.

Another key finding of our work is the association between *IL11^+^* fibroblasts and nonresponse to anti–TNF-α in IBD with fibrosis acting as the linking bridge. Analysis of the integrated bulk RNA-seq dataset based on CIBERSORTx (36) indicated that *IL11^+^* fibroblasts were significantly enriched in nonresponders. Interestingly, this effect was not observed in VDZ nonresponders, suggesting a specific association between *IL11^+^* fibroblasts and nonresponse to anti–TNF-α therapy. Moreover, enrichment analysis and experimental validation confirmed that DEGs of *IL11^+^* fibroblasts and genes in stromal-M13 are linked to ECM formation; meanwhile, nonresponders also showed elevated expression of genes involved in synthesizing ECM components like collagen and glycoproteins. Consistent with our findings, Smillie et al. (24) found that IAFs are already significantly enriched in the pretreatment intestinal tissues of anti–TNF-α nonresponders, potentially collaborating with inflammatory monocytes and DCs to mediate anti–TNF-α nonresponse via the oncostatin M/oncostatin M receptor (OSM/OSMR) signaling pathway. Previous studies have also associated *IL11* and *IL13RA2* with nonresponse (9, 34). Regarding the role of IL-11 in intestinal fibrosis, a recent study confirmed its significant enrichment in the fibrostenotic intestine, where it is primarily secreted by fibroblasts and demonstrates profibrotic effects in vitro, identifying it as a potential biomarker for predicting intestinal fibrotic stenosis in CD (51). From a broader perspective, *IL11^+^MMP1^+^CXCL8^+^IL7R^+^* inflammatory myofibroblasts have been demonstrated to potently promote fibrosis and scarring across diverse disease settings (49). As for the link between intestinal fibrosis and nonresponse to anti–TNF-α in IBD, although a positive correlation has been previously identified (10), the pathogenic cells potentially driving this effect were not identified. Therefore, building upon prior research, we established a connection between *IL11^+^* fibroblasts, fibrosis, and nonresponse to anti–TNF-α. The precise mechanisms through which fibrosis leads to nonresponse remain to be fully elucidated but may involve the engagement of non–TNF-dependent inflammatory pathways like IL-1– or TWEAK-related pathways (52, 53), as well as the persistence of fibrosis sustained by noninflammatory elements (e.g., mechanical stress, intestinal microbial components) (54–56).

Based on aforesaid discoveries, we proceeded to construct a predictive model for nonresponse to anti–TNF-α. Through SHAP, survival analysis, and IHC and other staining experiments, we further demonstrated the pivotal role played by *IL11* in this model. Furthermore, when compared with models established in previous studies (17, 18, 34), our model demonstrated superior performance. Notably, IL-11 was markedly upregulated in fibrotic lesions of nonresponders and closely correlated with anti–TNF-α treatment outcome in patients with CD, providing biological and clinical significance to the predictive model. Several previous studies, through meta-analyses or retrospective clinical cohorts, have identified associations between genetic markers (e.g., *FCGR3A* polymorphism) (57), patient lifestyle factors (e.g., smoking, overweight) (58, 59), and the activity of inflammation-related pathways (e.g., NF-κB, JAK/STAT3 pathways) (60) with response to anti–TNF-α therapy in IBD, albeit with modest significance. Moreover, these studies did not further incorporate the aforementioned indicators into the development of predictive models for treatment efficacy.

Building on the identification of nonresponse-associated genes including *IL11*, we constructed a predictive model for anti–TNF-α efficacy that also integrates other fibrosis- and inflammation-related genes, thereby conferring biological plausibility on our model. TWIST1, a basic helix-loop-helix transcription factor belonging to the TWIST family, is broadly expressed in fibrotic diseases (61). Our previous research identified TWIST1 as a critical transcription factor for the activation of intestinal fibroblasts in CD, particularly *FAP^+^* fibroblasts, which cooperates with TGF-β to promote the release of fibronectin, α-SMA, and collagen I, thereby driving ECM production (26). Moreover, *TWIST1* is associated with cellular senescence and inflammation in UC, where its stable high expression suggests nonresponse to IFX or VDZ (62). Two other genes in the model, *IL13RA2* and *TNFRSF11B*, also play significant roles in inflammatory responses. *IL13RA2* has been validated as a significant biomarker for predicting primary nonresponse to anti–TNF-α therapy (63). A study in murine dextran sodium sulfate–induced colitis models had demonstrated its high expression in epithelial cells, where it contributes to IBD pathology by inhibiting goblet cell function and impairing post-injury epithelial repair (64). *TNFRSF11B* is enriched in the blood of patients with sepsis–acute respiratory distress syndrome (sepsis-ARDS), mediates vascular endothelial dysfunction in inflammation, and represents a potential prognostic marker for sepsis-ARDS (43). Interestingly, *IL13RA2*, *TNFRSF11B*, and *IL11* have been confirmed not only as specific marker genes for IAFs in UC but are also closely associated with resistance to anti–TNF-α (24). In summary, our multi-gene model can effectively predict nonresponse to anti–TNF-α in patients with IBD and further underscores the intertwined roles of fibrosis and inflammation.

Our study has several limitations. First, the bulk RNA-seq datasets utilized employed varying definitions of nonresponse and consisted predominantly of samples from patients with UC, although intestinal fibrosis is more prevalent and clinically consequential in CD (65), limiting the generalizability of our findings and predictive model. Additionally, as the scRNA-seq dataset was stratified by inflammation rather than fibrosis, we had to infer the association between *IL11^+^* fibroblasts and fibrosis indirectly, for example through enrichment analysis. Thus, more direct evidence is required to further validate this association. Future studies are essential to address the current gap in scRNA-seq data capturing nonresponse to biologics in IBD and to elucidate the precise causal mechanisms linking intestinal fibrosis to anti–TNF-α nonresponse through more robust experimental and clinical evidence (e.g., validation at the protein level or larger patient cohorts), which were relatively lacking in our study. In parallel, gut microbiota such as *Mucispirillum schaedleri* and *Ruminococcus* have been shown to influence fibroblast activation and play a significant role in intestinal fibrosis (8, 66). The molecular basis underlying this profibrotic effect has also been explored, with studies highlighting the critical roles of bacteria-derived LPS, cyclic GMP/AMP, and DPP4, as well as TLR4 and OTUD3 expressed by intestinal fibroblasts (67–69). Meanwhile, genera including *Bifidobacterium*, *Morganella*, and *Enterococcus* have been used to predict response to IFX in patients with CD (70). Therefore, whether dysbiosis contributes to nonresponse to biologic therapies in CD through the activation of specific fibroblast subclusters merits further investigation.

In conclusion, our work reveals a close link between *IL11^+^* fibroblasts, fibrosis, and nonresponse to anti–TNF-α and leverages this to construct a multi-gene model for predicting anti–TNF-α treatment efficacy in IBD. Therefore, our study positions *IL11^+^* fibroblasts not only as the basis for a predictive model to guide precision therapy but also as a promising therapeutic target to overcome anti–TNF-α nonresponse in IBD.

## Methods

### Sex as a biological variable.

Both male and female patients were included in this study. Sex was not considered as a biological variable because the study was not designed to detect sex-specific differences.

### Collection and processing of public scRNA-seq and bulk RNA-seq datasets.

The scRNA-seq dataset GSE134809 (16) was downloaded from NCBI’s Gene Expression Omnibus (GEO), and SCP259 and SCP1884 (23, 24) were obtained from Single Cell Portal (SCP). All 3 datasets originated from intestinal tissues. Then, these datasets were integrated into a new dataset comprising samples from 57 patients with CD, 18 patients with UC, and 25 healthy individuals. Bulk RNA-seq datasets also derived from intestinal tissues were downloaded, including GSE16879, GSE12251, GSE23597, GSE212849, and GSE73661 from NCBI’s GEO (17, 33–35, 44), as well as E-MTAB-7914 from Biostudies (37). GSE16879, GSE12251, GSE23597, and GSE212849 were integrated for exploratory analysis and predictive model construction, encompassing a total of 200 patients with IBD treated with IFX or GLM, including 114 nonresponders and 86 responders. GSE73661, enrolling 23 patients with UC treated with IFX including 15 nonresponders and 8 responders, was utilized as a test set for the predictive model. E-MTAB-7914 (37), including 62 patients with IBD treated with VDZ (35 responders and 27 nonresponders), was included in our supplementary analysis of the association between *IL11^+^* fibroblasts and nonresponse. Our study prioritized obtaining normalized microarray data. If normalized data were unavailable, the RMA method from R package affy (v1.84) was applied to normalize raw data.

### Quality control of scRNA-seq data.

The raw count matrix of the integrated scRNA-seq dataset was filtered using the R package Seurat v5.3.0 (71): Cells with fewer than 500 or more than 6,000 genes, UMI counts below 500 or exceeding 30,000, mitochondrial gene content over 20%, or ribosomal gene content over 25% were removed. Doublet identification was performed using the R package DoubletFinder (v2.0.6). Doublets were detected with pN = 0.25, pK = 0.09, and an expected doublet rate of 4%. To further eliminate ambient RNA contamination, R package decontX (v1.4.0) was applied to estimate cell-specific contamination levels based on cluster information. Cells with a contamination fraction greater than 0.1 were excluded from downstream analyses. After quality control, a total of 505,578 cells were retained, including 96,139 from normal intestinal samples, 286,897 from noninflamed areas of IBD intestines, and 122,542 from inflamed areas of IBD intestines. Finally, count data from the filtered cells were normalized using the NormalizeData function in Seurat.

### Dimension reduction, clustering, annotation, and DEG identification.

The top 2,000 highly variable genes were selected using the FindVariableFeatures function; scaled using the ScaleData function; and variation sources from total counts, mitochondrial, erythrocytic, and ribosomal gene percentage were regressed out. Principal component analysis was performed on these highly variable genes, with the top 20 principal components selected for dimensionality reduction. Batch effects were corrected using the R package harmony (v1.2.1). An appropriate resolution parameter was determined with the R package clustree (v0.5.1), followed by cell clustering using the FindNeighbors and FindClusters functions in Seurat. Nonlinear dimensionality reduction was performed via UMAP using the RunUMAP function. Two-stage annotation was finally conducted: initial unsupervised clustering was used to annotate major intestinal cell types (B/plasma cells, T cells, stromal cells, epithelial cells, myeloid cells, and endothelial cells) based on canonical markers. Subsequently, stromal and myeloid cells underwent further unsupervised clustering and annotation as required. The proportion of each subcluster relative to the total cell count was calculated for both annotation stages. Dot plots were used to visualize the log-normalized expression levels of marker genes. Canonical or signature marker genes for stromal cells and myeloid cells are in Figure 1F and Supplemental Figure 1C. Additionally, DEGs of *IL11^+^* fibroblasts were identified using the FindMarkers function, retaining only upregulated genes with adjusted *P* value less than 0.05 and average log_2_ fold-change greater than 4.

### Identifying cellular modules via CoVarNet.

To uncover co-occurrence and cooperative patterns within the multicellular ecosystem of IBD intestinal tissue, CoVarNet was applied for comprehensive analysis of the scRNA-seq data. CoVarNet is a computational framework for identifying core cell clusters and constructing co-occurrence CM networks from cell frequency matrices (27). First, the frequency matrix of all annotated subclusters was computed and subjected to *z* score normalization to mitigate bias from differences in subcluster size. Nonnegative matrix factorization was then applied to the frequency matrix. The optimal number of modules was determined as 7 based on cophenetic correlation coefficients across different factorization ranks, and the feature and coefficient matrices were extracted. CM networks were constructed using the cm_network function from CoVarNet, integrating cooccurring CMs identified by Pearson-correlated edges (*r* ≥ 0.3, FDR < 0.05) and including up to 8 cells per module to generate both global and CM-specific networks. Visualization of cell-cell communication within CM01 was performed using R package igraph (v2.1.4).

### Analysis of cell-cell interaction.

The internal communication network within CM01 was first constructed using the R package CellChat (v1.6.1) (29). The netVisual_heatmap function was used to visualize the relationships and interaction strengths among cell subclusters in this module, confirming the central role of *IL11^+^* fibroblasts and identifying *S100A8/9^+^* monocytes and *FCGR3A^+^* monocytes as closely interacting partners. Subsequently, the R package nichenetr (v2.2.0) (30) was utilized to infer ligand-receptor pairs and downstream target genes between *IL11^+^* fibroblasts and *S100A8/9^+^* monocytes/*FCGR3A^+^* monocytes. *IL11^+^* fibroblasts were defined as sender cells; *S100A8/9^+^* monocytes and *FCGR3A^+^* monocytes as receiver cells. DEGs in receiver cells between inflamed and healthy control groups were used as the gene set of interest for NicheNet analysis. Finally, to analyze signaling pathway activity across cell types within CM01, the run_mlm function from the R package decoupleR (v2.9.7) (45) was employed. This function fits the normalized gene expression matrix to pathway-gene weight networks from PROGENy (28) and calculates the regression coefficient t-value for each pathway in each subcluster as its pathway activity score.

### Identifying gene modules by high-dimensional weighted gene coexpression network analysis.

High-dimensional weighted gene coexpression network analysis is a method for analyzing high-dimensional gene expression data such as scRNA-seq data. We employed the R package hdWGCNA (v0.4.7) (31) following its standard workflow. Cells were first grouped based on sample origin and cell type. The MetacellsByGroups function was used to construct metacells (k = 25) to reduce data sparsity and enhance the signal-to-noise ratio of coexpression signals. The gene expression matrix of the stromal cell cluster was extracted; then the analysis proceeded by using the TestSoftPowers function to determine the optimal soft power threshold (power = 7) and the ConstructNetwork function to build the gene coexpression network. kME and harmonized module eigengenes were calculated to evaluate gene-module correlations. To further explore the correlation of each gene module with inflammatory or fibrotic phenotypes and their differential expression between inflamed/noninflamed groups, module trait correlation and differential module eigengene analysis were also conducted.

### Cell deconvolution in bulk RNA-seq data.

CIBERSORTx (https://cibersortx.stanford.edu/) was used to estimate the composition of different intestinal cell types in bulk RNA-seq data (36). The cell subclusters from the previously analyzed scRNA-seq dataset were used as the input signature for the deconvolution operation of the integrated bulk RNA-seq dataset. Within each cluster, 100 cells were sampled. If the total number of cells in a subcluster was less than 100, all cells were selected. Differences in cell composition between the nonresponse and R groups were visualized after calculation. Subsequently, the R package Boruta (v8.0.0) (38) was applied to these identified cell types to filter the most associated cells with nonresponse to anti–TNF-α.

### DEG identification in bulk RNA-seq data.

To identify DEGs closely associated with nonresponse, bulk RNA-seq data were processed using R package limma (v3.62.1) (40). The lmFit function was used to fit a linear model for the transformed expression values of each gene, contrasts of interest were defined using the makeContrasts function, and empirical Bayes moderation was applied via the eBayes function. DEGs were identified with FDR less than 0.05 and |log fold change| greater than 1.

### Functional annotation and enrichment analysis.

GO and KEGG pathway enrichment analyses were conducted for DEGs of *IL11^+^* fibroblasts in scRNA-seq data, DEGs between groups in bulk RNA-seq data, and genes of stromal-M13 using the R package clusterProfiler (v4.14.4) (72). For scRNA-seq data, inflammation score or ECM signature scores were calculated for total intestinal cells, stromal cells, and myeloid cells using AddModuleScore function. The gene sets used to calculate inflammation and ECM score were obtained from published research (25) and GO:0030198 from Gene Ontology Resource (https://www.ebi.ac.uk/QuickGO/term/GO:0030198), respectively. For bulk RNA-seq data, ssGSEA was conducted via R package GSVA (v2.0.5) (73), using DEGs of *IL11^+^* fibroblasts in scRNA-seq analysis, genes of stromal-M13, collagen/glycoprotein/proteoglycan-associated gene sets from published literature (39), and the previously mentioned ECM gene set as input.

### Identification of signature genes for model construction.

Candidate genes were from the intersection of *IL11^+^* fibroblast DEGs, genes of M13, and DEGs in the integrated bulk RNA-seq dataset (Supplemental Table 1). To identify the optimal gene combination for model construction from these candidates, LASSO, random forest, and SVM were employed based on the R package caret (v7.0.1) (74). For LASSO, λ was adjusted to ensure appropriate specificity and sensitivity, and the gene combination corresponding to the minimum cross-validation error was selected. For random forest and SVM, the gene combinations yielding the highest accuracy were selected. The 5-fold cross-validation was employed across all 3 screening methods mentioned above. Signature genes were defined as the set of genes common to these 3 algorithms (Supplemental Table 2). Pearson’s correlations among these signature genes were calculated, and their individual AUC values were assessed by R package pROC (v1.18.5).

### Model construction and comparative evaluation.

After screening out the signature genes, 13 machine learning modeling algorithms including KNN, random forest, AdaBoost, Extra Trees, SVM, logistic regression, LDA, QDA, naive Bayes, LightGBM, Gradient Boosting, XGBoost, and Decision Tree were applied in Python to build prediction models based on the identified signature genes, with Bayesian optimization employed during model training under a 3-fold cross-validation setting. The model achieving the highest AUC value was selected as the final model. The training set (*n* = 140) and validation set (*n* = 60) were both randomly derived from the integrated bulk RNA-seq dataset from NCBI’s GEO including GSE16879, GSE12251, GSE23597, and GSE212849. The independent cohort GSE73661 (*n* = 23) served as the test set. To interpret the contribution of each key gene to the final model, SHAP analysis was performed using the Extra Trees classifier. All samples in the validation dataset were included in the SHAP computation. SHAP values were calculated for the positive class in this binary classification task. To visualize and summarize feature importance, a SHAP bee-swarm plot was generated, illustrating both the magnitude and direction of each key gene’s contribution. Moreover, 3 previously published predictive models (17, 18, 34) were identified through a systematic literature review and were implemented in this study for comparative purposes. To ensure a fair comparison, all these models were evaluated using the same training and validation sets applied in the development of our model. Each model was trained following the procedures originally described in the respective publications. The resulting model parameters were subsequently applied to the validation set to assess and compare predictive performance.

### Histological section preparation.

Inflamed bowel samples had been obtained from 36 patients with CD before they received biologics treatment, including 13 responders and 23 nonresponders. Detailed clinical information of these patients is provided in Supplemental Table 3. Fresh intestinal tissues were fixed in 4% paraformaldehyde for 24 hours. Then, the tissue samples were processed through a standardized series of dehydration and clearing steps before being embedded in paraffin wax. Paraffin-embedded blocks of these intestinal tissues were then cut into 4 μm serial sections for H&E and Masson’s trichrome staining.

### IHC staining and quantification.

IHC staining for IL-11 was performed on 4% paraformaldehyde-fixed, paraffin-embedded human intestinal tissue sections. Sections were dewaxed, rehydrated, and subjected to antigen retrieval under appropriate antigen retrieval conditions. After blocking endogenous peroxidase activity with 3% hydrogen peroxide, sections were incubated with primary anti–IL-11 antibody (Invitrogen, PAS-36544) diluted at 1:200 overnight at 4°C. This was followed by incubation with HRP-conjugated secondary antibody and visualization using 3,3’-diaminobenzidine (DAB) chromogen. Counterstaining was carried out with hematoxylin to visualize nuclei. For quantitative analysis of IL-11 expression, 5 random fields from the mucosal or submucosal layers were captured per sample. The images were analyzed in Fiji using the Color Deconvolution plugin to measure the percentage of IL-11–stained area and the average OD measured for each field. The mean values from the 5 fields per sample were calculated for statistical analysis.

### Immunofluorescence staining and imaging.

Fresh tissue samples were fixed in 1% paraformaldehyde at 4°C overnight, dehydrated in 30% sucrose for 12 hours, embedded in OCT compound, and frozen at –80°C until use. Tissues were sectioned into 10 μm slices and rehydrated in PBS for 10 minutes. Permeabilization was performed by incubating sections in precooled methanol at –20°C for 30 minutes. Sections were then blocked with blocking buffer (Beyotime, P0260). The slides were incubated with the following primary antibodies diluted at 1:200 at 4°C overnight: anti–IL-11 (Invitrogen, PAS-36544) and anti-vimentin (Abcam, ab8978). After washing with PBS, sections were incubated with fluorochrome-conjugated secondary antibodies: goat anti-rabbit IgG (Abcam, ab150080) and goat anti-mouse IgG (Abcam, ab150115) diluted at 1:500 for 1 hour at room temperature. Finally, the sections were incubated with FITC anti-collagen I antibody (SouthernBiotech, 1310-02) at a dilution of 1:200 for 3 hours at room temperature. After antibody incubation and subsequent washes, sections were mounted with DAPI Fluoromount-G (SouthernBiotech, 0100-20) and covered with coverslips. Images were acquired using an Olympus FV4000 microscope and analyzed with Imaris software (v9.0.1). Quantitative analysis was performed on paired inflamed and healthy control samples (*n* = 5 per group). For each sample, 3 randomly selected fields were quantified, and the mean expression levels of IL-11 and collagen I were calculated according to their respective fluorescence-positive area in each field. Statistical significance was assessed using Wilcoxon’s rank-sum test. The colocalization of IL-11 and vimentin signals in inflamed intestinal tissue was assessed using ImageJ (NIH). For each image, rectangular regions of interest were drawn over representative areas, and fluorescence intensity profiles for both channels were generated using the Plot Profile function to evaluate spatial overlap of the signals.

### qPCR.

Paired fibrotic and nonfibrotic intestinal samples were obtained from 8 patients with CD undergoing surgery. Total RNA was extracted from these intestinal samples using TRIzol (Invitrogen), and cDNA was synthesized with SuperScript III cDNA Synthesis kit (Invitrogen). mRNA expression levels were measured using SYBR Green on a 384-well real-time PCR system (QuantStudio 6 Flex System, Applied Biosystems). Primers were either obtained from PrimerBank or designed based on previously published studies (see Supplemental Table 4 for primer sequences). Relative mRNA expression was quantified according to the 2^–ΔΔCt^ method.

### Univariable regression and survival analysis.

Univariable logistic regression was performed to evaluate the predictive value of IL-11_%Area and inflammation indicators for nonresponse to therapy. Models were fitted using the glm function, with treatment response (response/nonresponse) as the dependent variable and IL-11_%Area, CRP, ALB, and ESR as independent variables. ORs and corresponding 95% CIs were extracted using the R package broom (v1.0.8) and visualized in a forest plot. Additionally, we performed ROC curve analysis to compare the predictive performance of IL-11_%Area with that of clinical variables available in our CD cohort — including BMI, smoking status, CRP, ESR, and ALB — for response to anti–TNF-α. Univariable Cox proportional hazards models were applied to assess the association between these variables and the time to nonresponse after treatment, with occurrence of nonresponse defined as the event. HRs and corresponding 95% CIs were calculated and presented in a forest plot. Furthermore, patients were stratified into high- and low-expression groups based on the median value of IL-11_%Area. Differences between these 2 groups were evaluated using the Kaplan-Meier method, and survival curves were plotted with the ggsurvplot function from the R package survminer (v0.5.0). The statistical significance of survival difference was determined by the log-rank test.

### Statistics.

Statistical analyses in our study included 2-sided, 2-tailed *t* test; Wilcoxon rank-sum test; log-rank test; Fisher’s exact test; Pearson’s correlation; and Spearman’s correlation. For multiple comparisons, FDR was controlled using the Benjamini-Hochberg procedure. All statistical analyses were performed using R or GraphPad Prism software (v10.1.2), with a *P* value of less than 0.05 considered statistically significant.

### Study approval.

The collection and use of all clinical biospecimens in this study were approved by the local ethics committee of Ruijin Hospital Affiliated to Shanghai Jiao Tong University School of Medicine (approval 2023-292). Written informed consent and patient assent were obtained from all enrolled patients prior to surgery and sample collection. Additionally, no patients or members of the public were involved in the study design, implementation, or analysis.

### Data availability.

The public scRNA-seq and bulk RNA-seq datasets included in this study have been comprehensively listed in *Collection and processing of public scRNA-seq and bulk RNA-seq datasets* in the Methods. The corresponding raw data for individual data points in the figures are provided in the Supporting Data Values file.

## Author contributions

Y Zhang, DZ, and YG conceived and designed the research and supervised the studies. WL conducted analyses. WL and JW performed the experiments. YT, QY, Y Zhou, ZZ, and HZ were involved in interpretation. WH wrote the manuscript. WL, JW, Y Zhang, DZ, and YG revised the manuscript. All authors read and approved the final manuscript. The order of the co–first authors’ names was assigned on the basis of the academic contribution of each author.

## Conflict of interest

The authors have declared that no conflict of interest exists.

## Funding support

- National Natural Science Foundation of China (82500624 to Y Zhang).
- National Natural Science Foundation of China (82341221 to DZ).

## Supplementary Material

Supplemental data

Supporting data values

## Version 1 04/23/2026

In-Press Preview

## Version 2 07/08/2026

Electronic publication
