# Supplementary material for: IL11+ fibroblasts are implicated in nonresponse to anti–TNF-α via fibrosis in inflammatory bowel disease
Source: JCI Insight. 2026 Apr 23;11(13):e198895. doi: 10.1172/jci.insight.198895 (PMC13460814; doi:10.1172/jci.insight.198895)
Supplement: Supplementary file 1 [file jciinsight-11-198895-s014.pdf]

Supplemental figures

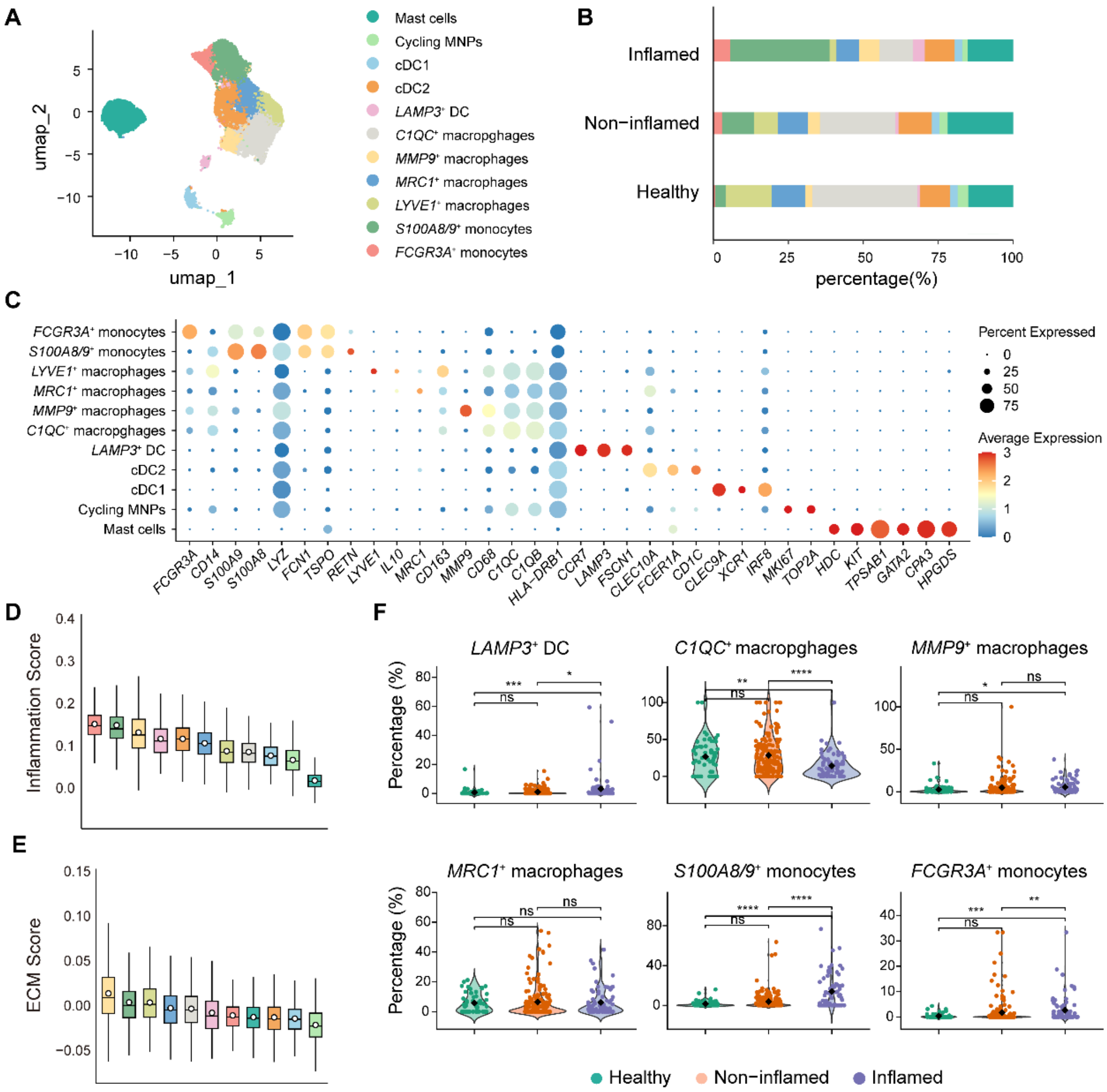

Supplemental Figure 1. Myeloid cell subclusters in the inflamed intestine of IBD. (**A**) UMAP plot showing 11 myeloid cell subclusters from the integrated scRNA-seq dataset, including 4,141 cells from the healthy control group, 10,562 from the non-inflamed group, and 18,827 from the inflamed group, totaling 33,530 cells.

(**B**) Bar graph showing the proportions of 11 myeloid cell subclusters across the inflamed, non-inflamed, and healthy control groups.

(**C**) Dot plot showing the characteristic marker genes for each myeloid cell subcluster. Dot color and size respectively represent average expression level and expression percentage.

(**D-E**) Box plots showing the inflammation scores (**D**) and ECM scores (**E**) for each myeloid cell subcluster. Median, quartiles, and range are shown.

(**F**) Proportion of *LAMP3*$^{+}$ DC, *C1QC*$^{+}$ macrophages, *MMP9*$^{+}$ macrophages, *MRC1*$^{+}$ macrophages, *S100A8/9*$^{+}$ monocytes, and *FCGR3A*$^{+}$ monocytes in the healthy control, non-inflamed, and inflamed groups. Statistical significance was determined by Wilcoxon rank-sum test (ns: $p > 0.05$; *: $p \leq 0.05$; **: $p \leq 0.01$; ***: $p \leq 0.001$;

****: $p \leq 0.0001$).

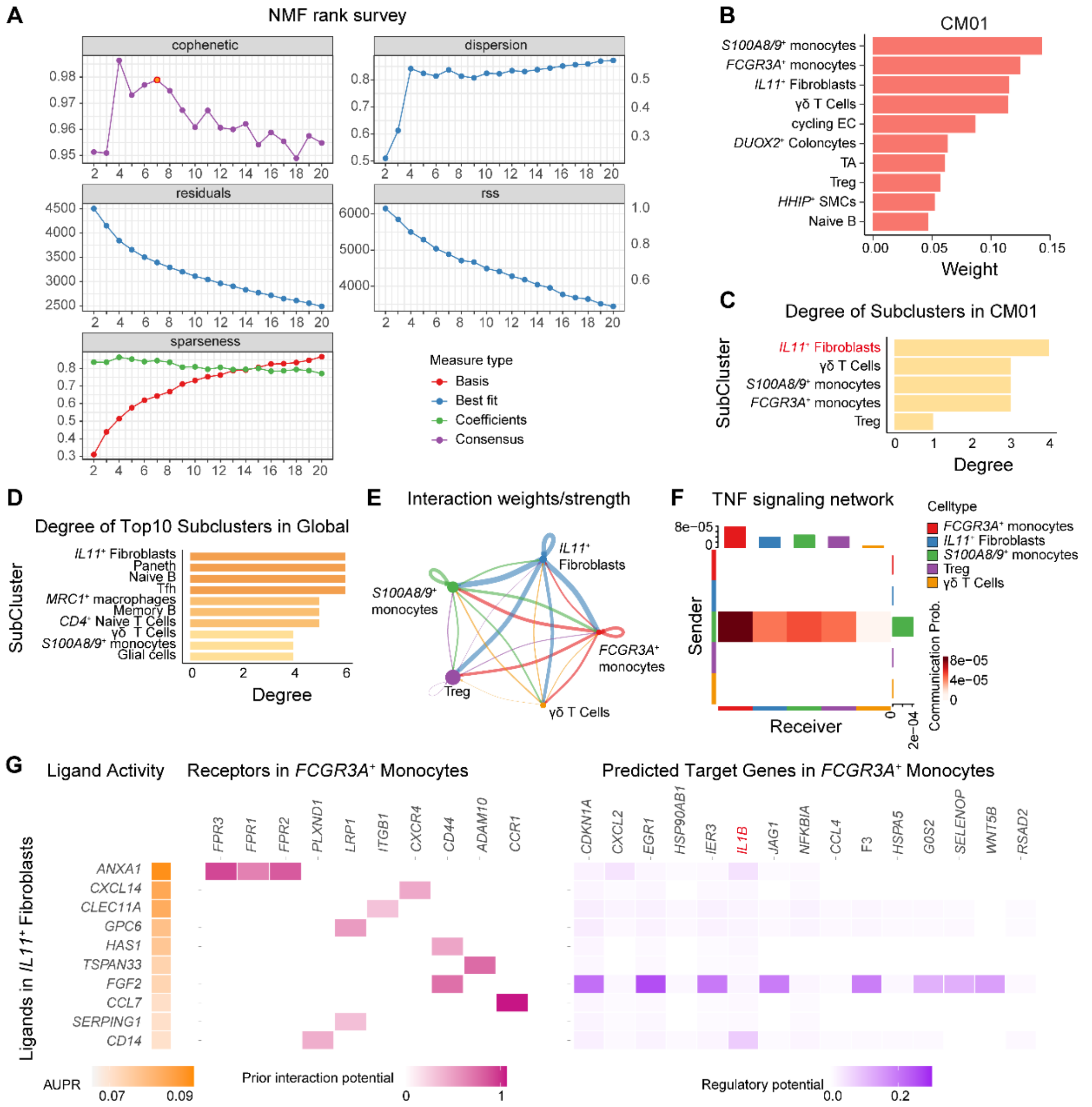

Supplemental Figure 2. An *IL11*⁺ fibroblast-centric CM and its internal interaction network.
(**A**) Line graphs showing the NMF rank survey in CoVarNet analysis. Parameters displayed include cophenetic, dispersion, residuals, residual sum of squares (RSS), and sparseness.
(**B**) Bar graphs showing the module weight calculated by NMF of subclusters in CM01.
(**C-D**) Bar graph showing the degree (number of associations with other subclusters in the module) of subclusters in CM01 (**C**) and the top 10 subclusters in global interaction network (**D**).
(**E**) Network graph showing the interaction among core subclusters in CM01. Line number represents the number of associations, and line thickness represents the strength of associations.
(**F**) Heatmap showing the interaction strength in TNF signaling pathway among the five core cell subclusters of CM01.
(**G**) Heatmap displaying the top-ranked ligand-receptor pairs inferred by NicheNet for

IL11^{+} fibroblasts regulating *FCGR3A*$^{+}$ monocytes. The left panel shows the activity of top-ranked ligands from *IL11*$^{+}$ fibroblasts and their interaction strength with potential receptors on *FCGR3A*$^{+}$ monocytes; the right panel shows the downstream target genes in *FCGR3A*$^{+}$ monocytes. The meaning represented by different grid colors is shown at the bottom of the figure.

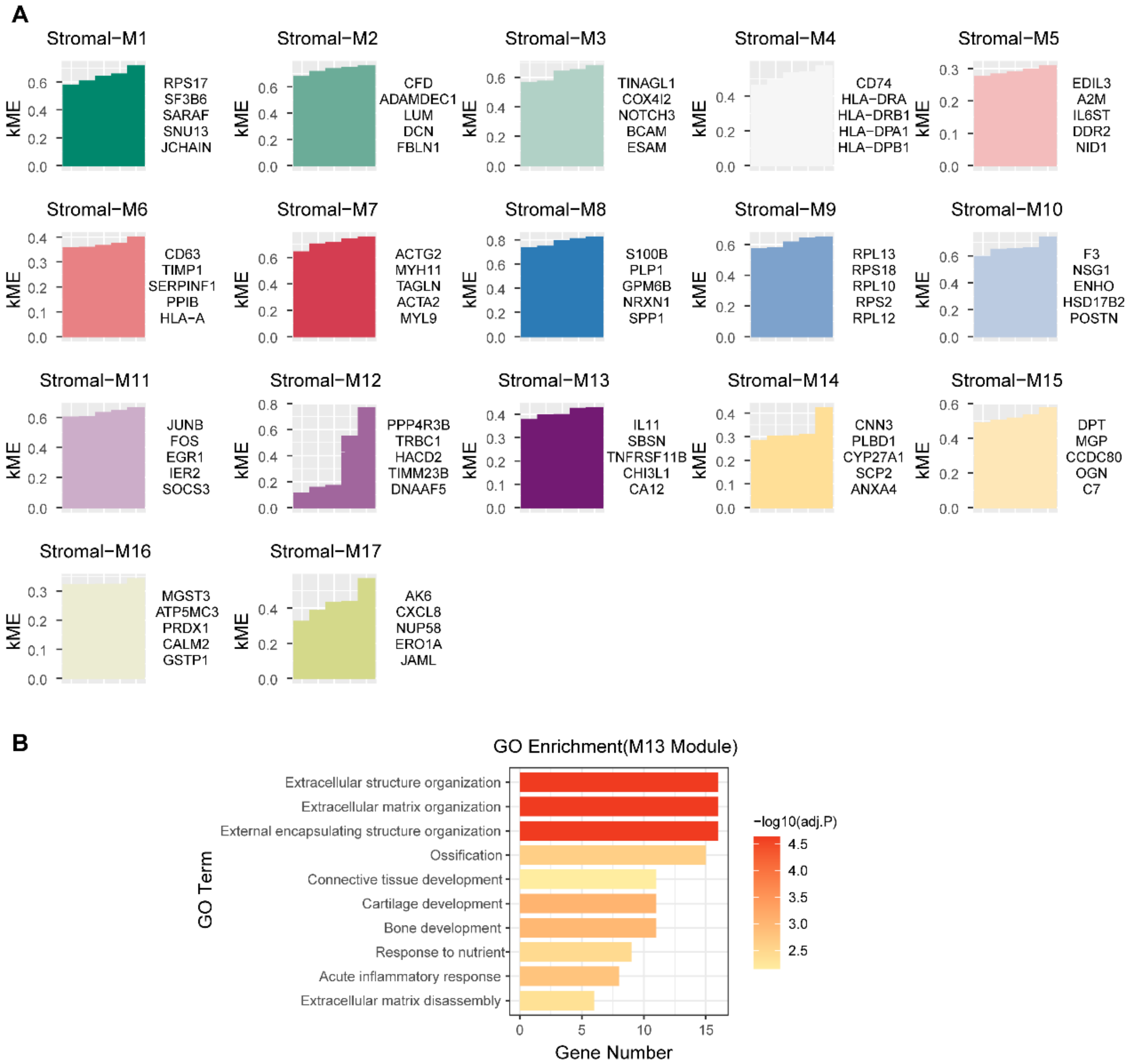

Supplemental Figure 3. hdWGCNA reveals characteristic stromal-associated gene modules in IBD.

(**A**) Ranking and kME values of the top5 genes within stromal-M1-17.

(**B**) Representative GO enrichment analysis for the genes of stromal-M13. A hypergeometric test was conducted using FDR-adjusted p values.

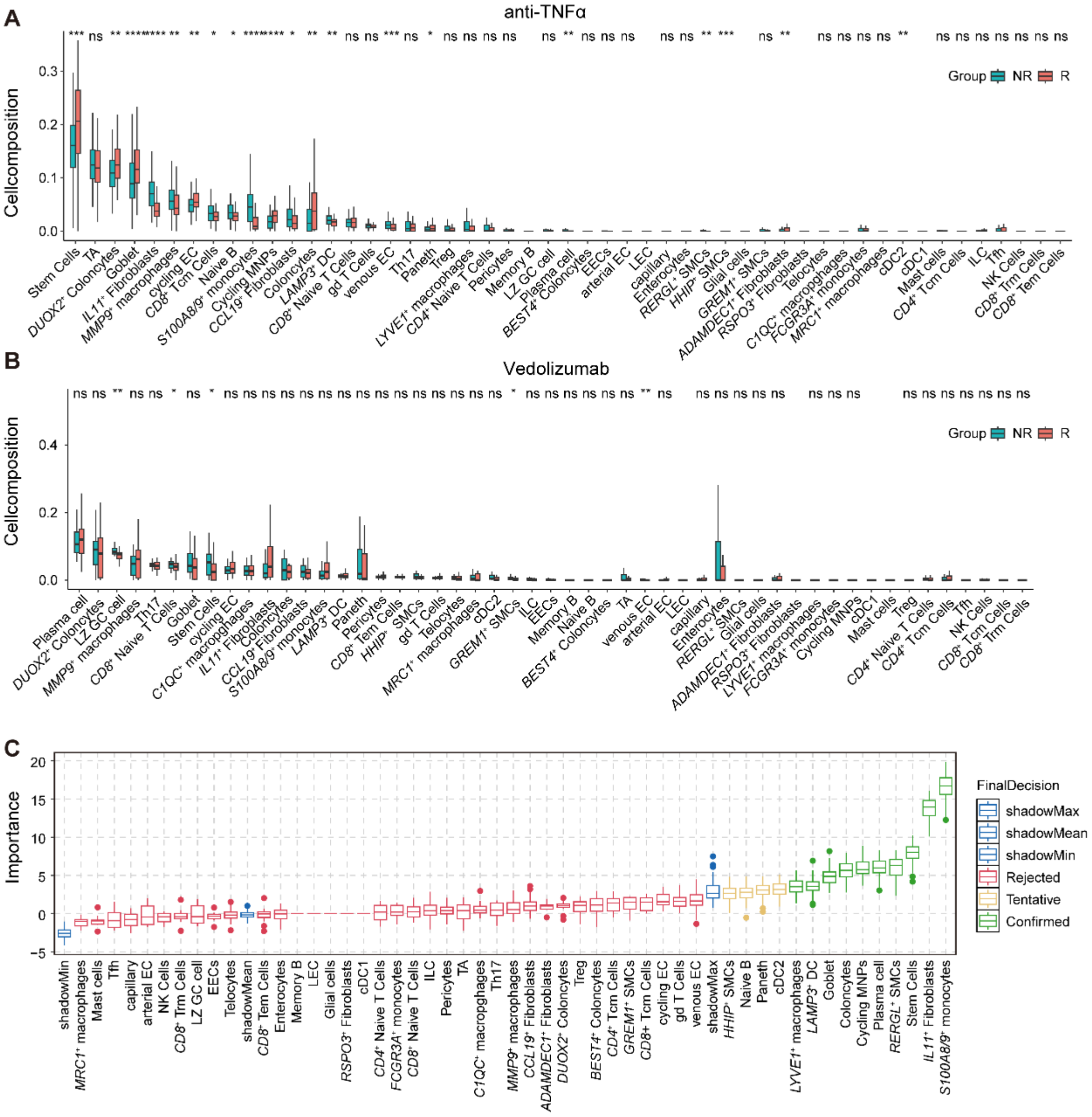

Supplemental Figure 4. NR to anti-TNFα is potentially relative to *IL11*⁺ fibroblasts.

(**A**) Box plots showing the stromal cell composition calculated by CIBERSORT in the integrated bulk RNA-seq dataset and its distribution differences between non-responders and responders. Median, quartiles, and range are shown. Statistical significance was determined by Wilcoxon rank-sum test (ns: $p > 0.05$; *: $p \leq 0.05$; **: $p \leq 0.01$; ***: $p \leq 0.001$; ****: $p \leq 0.0001$).

(**B**) Box plots showing the cellular composition calculated by CIBERSORT and its distribution differences between non-responders and responders. Median, quartiles, and range are shown. Statistical significance was determined by Wilcoxon rank-sum test (ns: $p > 0.05$; *: $p \leq 0.05$; **: $p \leq 0.01$).

(**C**) NR-associated importance ranking via Boruta of different cells identified by CIBERSORT.

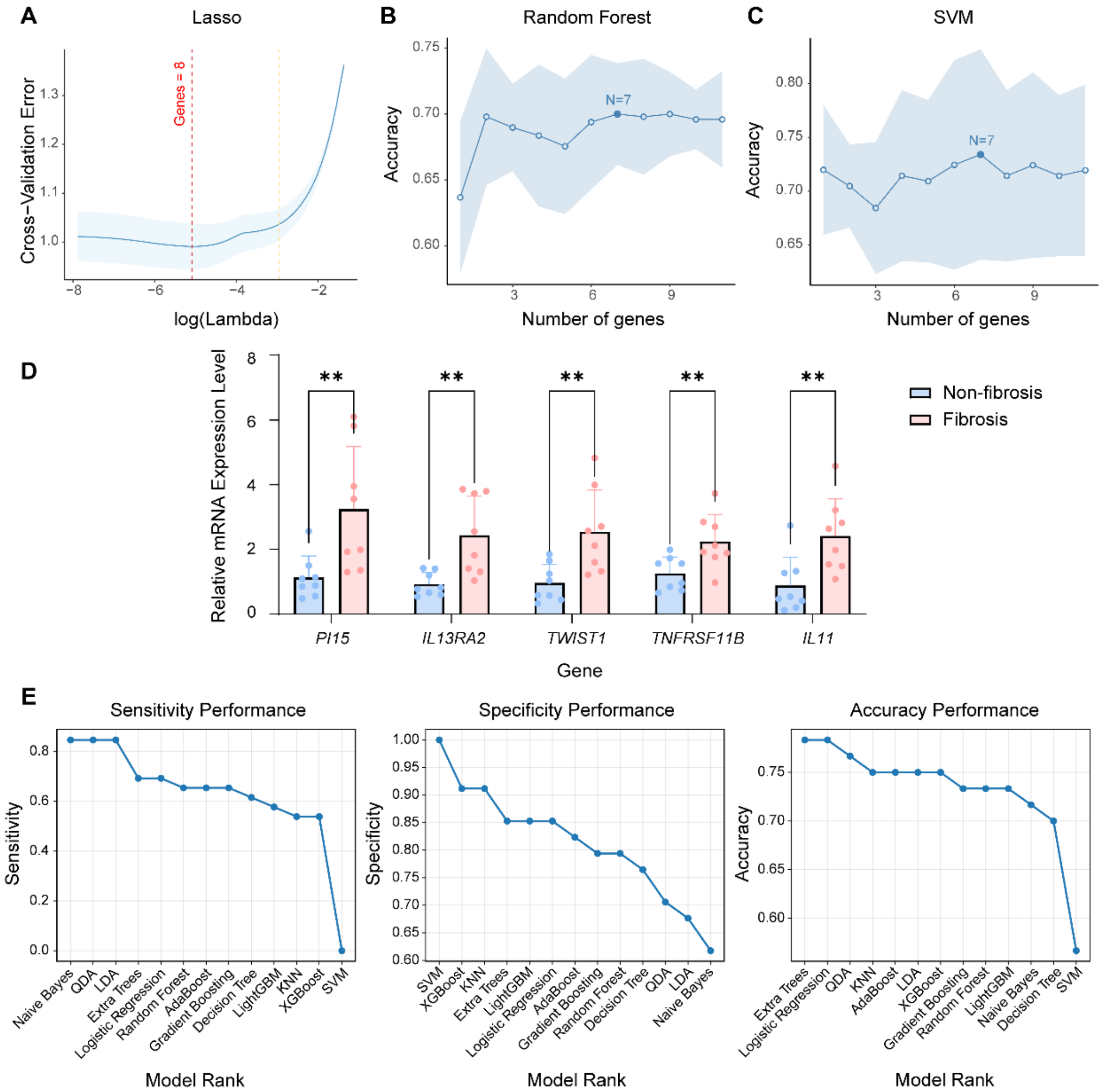

Supplemental Figure 5. Development of a predictive model for NR to anti-TNFα based on *IL11*$^+$ fibroblasts.

(**A-C**) The optimal number of key genes calculated by LASSO (**A**), RF (**B**) and SVM (**C**).

(**D**) Bar plots showing relative mRNA expression level of PI15, IL13RA2, TWIST1, TNFRSF11B, and IL11 in the paired fibrotic (n = 8) and non-fibrotic (n = 8) intestinal samples from eight CD patients. Data are presented as the mean ± SD. Statistical significance was determined by paired Wilcoxon's rank-sum tests (**: $p \leq 0.01$).

(**E**) Line graphs showing the sensitivity, specificity, and accuracy of the 13 models established by KNN, RF, AdaBoost, Extra Trees, SVM, Logistic Regression, LDA, QDA, Naive Bayes, LightGBM, Gradient Boosting, XGBoost, and Decision Tree.

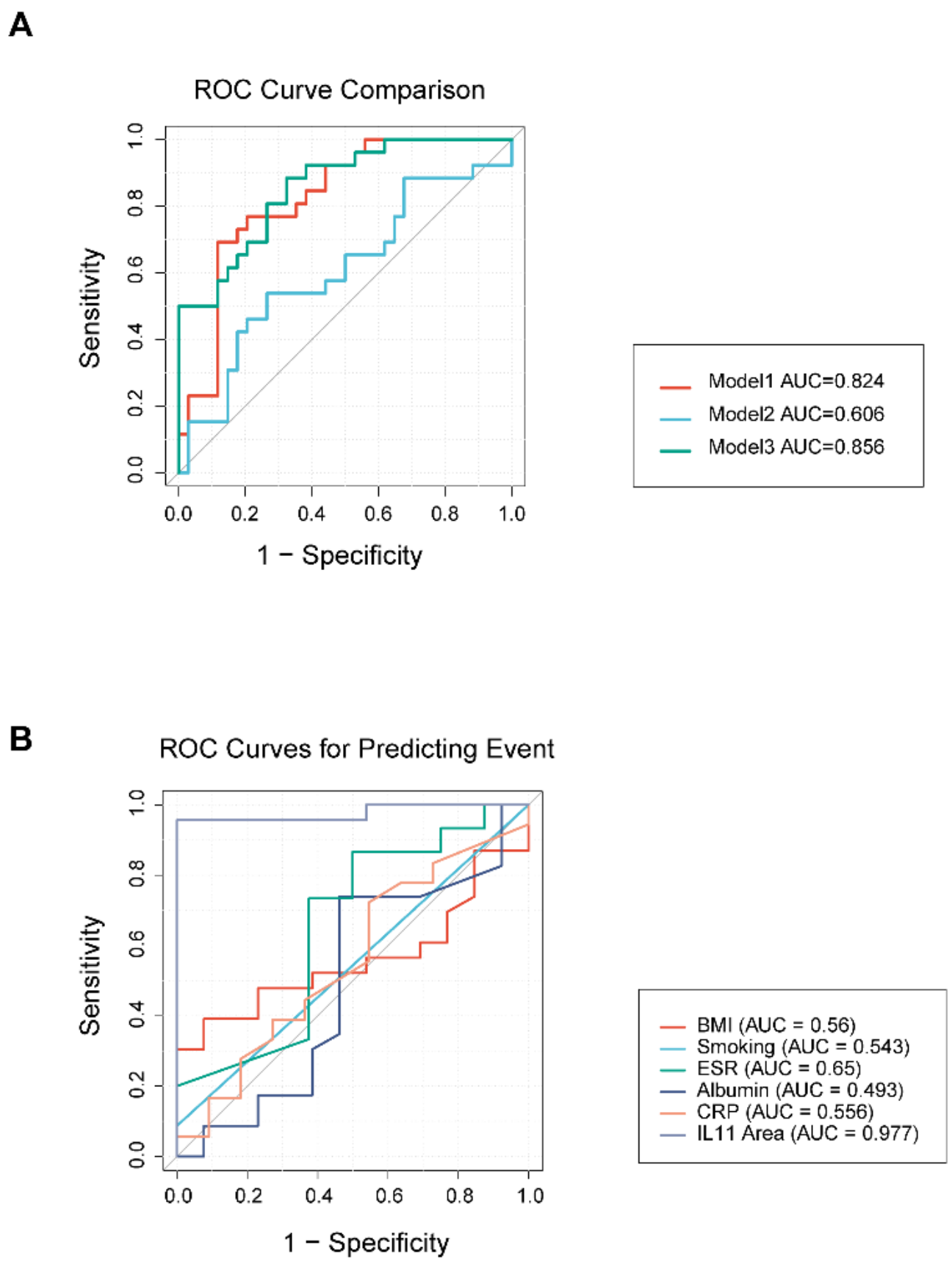

Supplemental Figure 6. Superior performance of the *IL11*+ fibroblast-based predictive model compared with previously published models and clinical indicators.

(**A**) ROC curves of Model1, 2, and 3 in the validation set. Corresponding model AUC values are listed on the right.

(**B**) ROC curves showing the predictive performance of BMI, Smoking, ESR, ALB, CRP, and IL11_%Area in the clinical cohort comprising 36 CD patients undergoing surgical resection. Corresponding indicator AUC values are listed on the right.

Supplemental tables

Supplemental Table 1. Signature genes of. 1L11_Fibro DEGs, M13 Module Genes, and bulk DEGs in Figure 5A.

Geneset	Signature genes
1L11_Fibro DEGs	*DCN, COL1A2, LUM, COL3A1, COL1A1, C1S, COL6A2, MMP2, COL6A1, RARRES2, CTSK, C1R, COL6A3, FBLN1, PCOLCE, IGFBP5, SERPINF1, NNMT, IGFBP4, SPON2, PTGDS, THY1, BGN, C11orf96, TGFBI, COL5A2, AEBP1, FSTL1, CXCL1, CHI3L1, PDPN, EFEMP2, IGFBP6, FGF7, COL12A1, CCL11, CTHRC1, ECM1, COL5A1, ANGPTL2, MXRA8, FBN1, TNC, ISLR, FKBP10, CXCL6, STEAP1, HTRA3, PTGES, PDGFRB, PDLIM4, FAM20C, MRGPRF, OLFML3, LOXL2, TNFAIP6, RCN3, ANTXR1, MASP1, CDH11, C3, FAP, LOXL1, COL7A1, WNT5A, CPXM1, THBS2, TNFRSF11B, TNFRSF12A, TDO2, MRC2, FIBIN, RAMP1, FBLN2, SPHK1, PTGS1, PRRX1, COL8A1, GLT8D2, GPX8, CLMP, BICC1, CRABP2, IL11, PRR16, TPST1, GUCY1A1, IL34, PTGFR, GLIS3, MMP1, MFAP2, PTHLH, LPAR1, PAPPA, FAM20A, TWIST1, TFPI2, SRPX2, WNT2, OLFM1, AKR1C1, EML1, VASN, TMEM158, ADAMTS2, LOX, MMP3, TMEM132A, CD248, LSAMP, HGF, SGIP1, BNC2, TSPAN11, LAMC3, RIPOR3, TCEAL7, GAS1, LTBP2, SBSN, PI15, LARP6, NFATC4, STC1, ACKR4, PAPLN, SDK1, HSD11B1, SSC5D, CXCL5, PRRX2, PODNL1, ROR2, S100A3, SNCAIP, CSF3, GALNT15, MEDAG, IL24, STRA6, SPOCK1, FNDC4, FAM110B, ARHGAP28, RASL12, SMOC1, IGDCC4, P4HA3, CCN4, GPR176, NID2, ADAM33, FNDC1, RP11-166D19.1, ADAMTS12, GLI3, IL13RA2, RP11-125O18.1, NTN1, SFRP2, CYP26B1, BDKRB1, DNM1, TFAP2C, ASPHD1, CLIC6, PTPRN, FDCSP, SORCS2, AOX1, BHLHE22, EGFL6, TGFB3, TWIST2, RGS4, VGLL3, FST, RP11-588K22.2, DIO2*
M13 Module Genes	*PDPN, ZCCHC17, SERINC2, CSMD2, CHI3L2, S100A2, CRABP2, RGS4, F5, PRRX1, RP1-79C4.4, FMO1, CHI3L1, IL10, C1orf115, RP11-400N13.3, CYP26B1, GNLY, CYTOR, IGKV1-12, IGKV1-9, IGKV2-24, STEAP3, KYNU, GALNT5, FAP, CHN1, DIRC1, IGFBP2, PTPRN, SLC19A3, MLPH, TWIST2, ANKRD28, GALNT15, COL7A1, CACNA2D3, ACKR4, MME, ECE2, NICOL1, FDCSP, CXCL5, CXCL3, EREG, CXCL13, ACSL1, ADAMTS12, IL7R, SLC1A3, LUCAT1, GLRX, PCSK1, TNFAIP8, SLC22A4, ATOX1, ELOVL2, CFB, UQCC2, HMGA1, SLC16A10, VNN1, SGK1, RP11-557H15.3, RP3-428L16.2, AGPAT4, TWIST1, INHBA, ASL, WNT2, EGFL6, GK, SRPX2, BGN, CSGALNACT1, SLC39A14, STC1, DUSP4, ANK1, PI15, CTHRC1, TNFRSF11B, CCN4, GLIS3, SEC61B, PRRX2, PTGES, ASS1, EGFL7, CD82,*

	TMEM132A, SYT7, P4HA3, MMP1, MMP13, ADAM12, PTHLH, FKBP11, C12orf75, HPD, RUBCNL, TMEM255B, PTGDR, IGHG2, IGHG1, IGHG3, IGHV1-24, APBA2, CA12, STRA6, ISG20, TNFRSF12A, SEZ6L2, ASPHD1, COTL1, RP11-863P13.3, ADORA2B, RASD1, CSF3, ABCC3, NME1, ARSG, WIPI1, FAM20A, CD300E, SPHK1, SLC16A3, TTC39C, CPXM1, SMOX, HCK, SULF2, TPM4, ARRDC2, SBSN, FPR1, IL11, MRAP, KCNJ15, RP11-525A16.4, SMOC1, PFKFB4, PHEX, MMP3, ETV4, LAMA1, RP1-90J20.8, AC004878.2, C2CD4A, IL24, GRIP2, MASP1, RP11-125O18.1, POPDC3, RP11-350J20.12, AMZ1, NRCAM, IL13RA2, FAM167A, LAMC3, MMP10, MUCL1, TEX29, IGHV3-66, RP11-426C22.4, TFAP2C, CTD-2545M3.8, TMEM190, FFAR3, DLG3-AS1, AMBP, ORM1, RNF175, AC024592.12, IL19, AC003092.1, NOTUM, SEPTIN3, ZNF295-AS1, ORM2, CPNE4, COL22A1, NELL1, RDH8, CFAP161, UCN2, RP11-64P14.7, RP11-993B23.3, RP11-495P10.6, LINC01050, PRL, RP11-18B16.2, LINC00922, RP11-417E7.1, XXbac-B33L19.4, C20orf141, AC005682.5, AC083884.8, D87007.1, GK-IT1, LL22NC03-N14H11.1, MT-TD, MT-TP, MT-TV, RP11-242C19.2, RP11-389C8.2, RP11-404P21.5, RP11-498E2.9, RP11-70J12.1, RP11-887P2.6, AC016722.2, AP000525.9, CTA-268H5.12, D86998.1, IGKV6D-41, RN7SL674P, RP11-183M13.1, RP11-554A11.4, RP11-810P8.1, RP11-82L18.2, RP5-916O11.2
bulk DEGs	*IL13RA2, PTGS2, STC1, WNT5A, IL11, PI15, TNFRSF11B, CSGALNACT1, IL6, LILRB2, MNDA, TFPI2, TWIST1, CTHRC1, IL24, CXCR2, FCN1, TNFAIP6, CXCL6, TREM1, S100A9, C5AR1, G0S2, PTX3, SELE, NCF2, VNN2, FCGR3A, SLC2A3, PKIB, SLC2A14, FCGR3B, THBS2, S100A12, APOBEC3A, FPR2, GPR84, PROK2, CSF3R, CMTM2, S100A8, MMP1, BCL2A1, SELL, CCL3, IL1B, AQP9, MMP3, HCAR3, CXCL5, IRG1, MCEMP1, CNTN3, RETNLB, KLK10, CLCA1, ANXA10, UGT2A3, CLDN8, DEFA5*

Supplemental Table 2. Gene combinations identified by LASSO, RF, and SVM respectively in Figure 5E.

Algorithm	Gene combination
LASSO	*TWIST1, STC1, PI15, CTHRC1, TNFRSF11B, IL11, MMP3, IL13RA2*
RF	*IL11, IL13RA2, TWIST1, PI15, IL24, TNFRSF11B, MMP1*
SVM	*IL11, PI15, IL13RA2, STC1, TWIST1, TNFRSF11B, IL24*

Supplemental Table 3. Clinical information of patients providing inflamed intestinal specimens, related to Figure 6, C-H.

Indicators	NR (n = 23)	R (n = 13)	P-value
**Age (y/o)**	44.48 ± 11.58	37.38 ± 10.69	0.07
**Gender**	Male (n = 15)<br>Female (n = 8)	Male (n = 9)<br>Female (n = 4)	1.00
**BMI (kg/m^2)**	20.33 ± 3.85	19.10 ± 1.73	0.20
**ESR (mm/h)**	10.80 ± 13.99	24.00 ± 27.39	0.26
**ALB (g/L)**	38.35 ± 5.90	38.77 ± 10.09	0.96
**CRP (mg/L)**	17.54 ± 46.31	7.74 ± 12.16	0.64
**Smoking history**	yes (n = 2)<br>no (n = 21)	yes (n = 0)<br>no (n = 13)	0.54

All continuous variables in this table are presented as mean ± SD. Statistical tests employed include: Welch's t-test (Age, BMI), Wilcoxon rank-sum test (ESR, ALB, CRP), and Fisher's exact test (Gender, Smoking history). y/o, years old.

Supplemental Table 4. Primer sequences for qPCR.

Gene	**Species**	**Forward primer**	**Reverse primer**
IL11	Homo sapiens	GGACCACAACCTGGATTCCCTG	AGTAGGTCCGCTCGCAGCCTT
IL13RA2	Homo sapiens	AACCTGGCATAGGTGTACTTCTTG	CACACTGTAATGCATGATCCAAGC
TNFRSF11B	Homo sapiens	CTGCGCGCTCGTGTTTC	ACAGCTGATGAGAGGTTTCTTCGT
TWIST1	Homo sapiens	AGCTACGCCTTCTCGGTCT	CCTTCTCTGGAAACAATGACATC
PI15	Homo sapiens	CAAGTACCGTCGTCCTACTCA	TCCGCTGAATCTAATTGTGCTT
GAPDH	Homo sapiens	GGAGCGAGATCCCTCCAAAAT	GGCTGTTGTCATACTTCTCATGG
